# β Boswellic Acid Blocks Articular Innate Immune Responses: An In Silico and In Vitro Approach to Traditional Medicine

**DOI:** 10.3390/antiox12020371

**Published:** 2023-02-03

**Authors:** Eloi Franco-Trepat, Ana Alonso-Pérez, María Guillán-Fresco, Miriam López-Fagúndez, Andrés Pazos-Pérez, Antía Crespo-Golmar, Susana Belén Bravo, Verónica López-López, Alberto Jorge-Mora, José P. Cerón-Carrasco, Ana Lois Iglesias, Rodolfo Gómez

**Affiliations:** 1 Musculoskeletal Pathology Group, Institute IDIS, Santiago University Clinical Hospital, 15706 Santiago de Compostela, Spain; 2 Centro Universitario de la Defensa, Universidad Politécnica de Cartagena, C/Coronel López Peña S/N, Base Aérea de San Javier, Santiago de La Ribera, 30720 Murcia, Spain

**Keywords:** osteoarthritis, chondrocytes, synoviocytes, ROS, TLR4, IL1, NLRP3

## Abstract

Osteoarthritis (OA) is hallmarked as a silent progressive rheumatic disease of the whole joint. The accumulation of inflammatory and catabolic factors such as IL6, TNFα, and COX2 drives the OA pathophysiology into cartilage degradation, synovia inflammation, and bone destruction. There is no clinical available OA treatment. Although traditional ayurvedic medicine has been using *Boswellia serrata* extracts (BSE) as an antirheumatic treatment for a millennium, none of the BSE components have been clinically approved. Recently, β boswellic acid (BBA) has been shown to reduce in vivo OA-cartilage loss through an unknown mechanism. We used computational pharmacology, proteomics, transcriptomics, and metabolomics to present solid evidence of BBA therapeutic properties in mouse and primary human OA joint cells. Specifically, BBA binds to the innate immune receptor Toll-like Receptor 4 (TLR4) complex and inhibits both TLR4 and Interleukin 1 Receptor (IL1R) signaling in OA chondrocytes, osteoblasts, and synoviocytes. Moreover, BBA inhibition of TLR4/IL1R downregulated reactive oxygen species (ROS) synthesis and MAPK p38/NFκB, NLRP3, IFNαβ, TNF, and ECM-related pathways. Altogether, we present a solid bulk of evidence that BBA blocks OA innate immune responses and could be transferred into the clinic as an alimentary supplement or as a therapeutic tool after clinical trial evaluations.

## 1. Introduction

The worldwide number of patients suffering from rheumatic diseases such as osteoarthritis (OA) equals the whole United States of America (USA) population. That is more than 300 million people that will suffer their whole life from daily chronic pain and disability with no option for an efficient treatment [1,2,3]. Currently, OA is defined as a complex heterogenic disease [4,5,6] with no clinical [7,8,9] or molecular [10,11,12] consensus on the disease pathophysiology. It is agreed to be a silent progressive degradative disease that hinders the early diagnosis of articular cartilage degradation, synovial inflammation [13,14], and/or bone alteration [15,16]. Then, biomechanical, metabolic, and aging-related alterations accumulate to the point that the whole joint space narrows due to cartilage destruction [17,18,19]. Chronic articular destruction activates inflammatory, catabolic, and pain pathways that lead to acute pain that might bring permanent disability [17,18,20]. Great efforts have been committed to different drug discovery approaches; pharmacomodulation [21], drug repurposing [22,23], and the re-discovery of natural products from traditional medicine, namely, the first Chinese medicine book *Hunagti Neiching* [24] and the Indian Ayurvedic medicine [25].

Indian traditional medicine has preserved a rich repertoire of antiarthritic remedies including Amalaki (*Embelia officinale*), Maricha (*Piper nigrum*), Turmeric (*Curcuma longa*) [26], and *Boswellia serrata* extract (BSE) mix [27]. Furthermore, several studies have shown that the Indian traditional remedy BSE mix inactivates multiple immune-related pathways [28]. Nonetheless, the lack of a normalized composition for the BSE mix [29,30] has caused several inconsistencies in the potential therapeutic effects on rheumatic diseases such as OA and Rheumatoid Arthritis (RA). This paradigm might change via the standardized BSE mix initiative Casperome [31]. Although most studies suggest that the BSE mix contains nuclear factor kappa-light-chain-enhancer of activated B cells (NFκB) inhibitors [32,33], other studies suggest that NFκB inhibition might indirectly be via 5-lipoxygenase [27,33] or reactive species of oxygen (ROS)-related processes [34,35]. Research on individual BSE mix components such as 11-Keto-β-Boswellic Acid (KBA) and Acetyl-11-Keto-β-Boswellic Acid (AKBA) is very limited; nevertheless, anti-inflammatory and anticatabolic effects were found consistently across the different in vitro, in vivo, and food supplement studies [33,36,37]. Interestingly, onlu 3% of the BSE mix studies have focused on the BSE mix component β Boswellic Acid (BBA), also known as beta-BA. Moreover, BBA was able to block cartilage loss and inflammatory factors in Toll-like Receptor 4 (TLR4)/Interleukin 1 Receptor (IL1R)-activated synovial explant tissues [27]. Nonetheless, little is known about the specific role of BBA on TLR4 or IL1R receptors.

The innate immune receptor TLR4 has not been fully explored as a therapeutic target in the clinical rheumatology field [38], nonetheless, its signaling crosstalks with IL1R [39,40,41] a well-explored therapeutic target for OA [42,43,44,45,46,47,48,49] and RA [50,51]. The degradation in the OA joint predominantly affects the cartilage and releases damage-associated molecular patterns (DAMPs) [17,52] that activate TLR4 signaling. In contrast, IL1R is activated by the accumulation of cytokine interleukin 1β (IL1β). The downstream signaling of both receptors meet in myeloid differentiation primary response 88 (MyD88), a molecule involved in the signal transduction and activation of NFκB [53]. The NFκB pathway modulates several OA-associated innate immune factors such as nitric oxide synthase 2 (NOS2), cyclooxygenase-2 (COX2), interleukin 6 (IL6), and matrix metalloproteinase 3 (MMP3) or 13 (MMP13) [54,55,56] that cause inflammation and tissue degradation. Remarkably, BBA has been shown to inhibit the NFκB pathway in osteoblasts [57], macrophages [32], and other cell types [58,59,60]. Nonetheless, there is a lack of data on BBA inhibition of NFκB in chondrocytes and cartilage.

In the present study, we selected the understudied BBA molecule from the BSE mix to finely elucidate the potential therapeutic effects on OA joint cells. Moreover, we made use of computational chemistry, a new current trend in drug discovery screening [61,62], to demonstrate the higher binding affinity [63] between BBA and TLR4 compared to other potential target candidates, as well as the ability of BBA to dock the TLR4 receptor in a passive and more advanced dynamic in silico model. Furthermore, we demonstrated that this antagonism of the TLR4 complex inhibited TLR4 and IL1R-associated innate immune responses in chondrocytes, osteoblasts, and synoviocytes isolated from OA patients. Moreover, we propose a mechanism of action to support the inhibitory effect of BBA on both receptors.

All in all, the use of BBA as a nutraceutical in clinical practice might be useful in the management of degradative joint processes observed in OA patients.

## 2. Materials and Methods

### 2.1. Docking Analysis

Molecular 3D structures of β boswellic acid (BBA), CLI-095 or TAK242, myristic acid, 2-OH myristic acid, 3-OH myristic acid, and lauric acid were obtained from the PubChem database [64], whereas NFκB (1SVC), IκB kinase (IKK) (3RZF), NLR family pyrin domain containing 3 (NLRP3) (6NPY), Toll-like Receptor 3 (TLR3) (3CIY), TLR4 (3VQ1), and IL1R1 (1ITB) biomolecular targets were obtained from protein data bank (PDB) [65]. Remarkably, Xu et al. deposited the IKK (3RZF) crystal [66] and resolved the active-site residues (Leucine 21 (Leu21), Glycine 22 (Gly22), Threonine 23 (Thr23), Valine 29 (Val29), Alanine 42 (Ala42), Lysine 44 (Lys44), Methionine (Met96), Glutamine (Glu97), Tyrosine 98 (Tyr98), Cystine 99 (Cys99), Gly102, Asparagine 103 (Asp103), Glu149, Asparagine 150 (Asn150), Isoleucine 165 (Ile165), and Aspartic Acid 166 (Asp166) in presence of the Cmpd1 inhibitor known as (4-{[4-(4-Chlorophenyl) pyrimidin-2-Yl] amino} phenyl) [4-(2-Hydroxyethyl) piperazin-1-Yl] methanone. Similarly, Mao et al. deposited the NLRP3 (6NPY) crystal [67] that resolves the adenosine diphosphate (ADP) binding site and has successfully been used for virtual screening simulations [68,69]. All protein database (PDB) crystallographic structures were refined with the protein preparation wizard module implemented in the Schrödinger suite [70]. A grid was generated at the ligand sites to attempt a molecular docking using the extra-precision (XP) scoring functions implemented in Glide [71,72]. Binding energies were computed at the molecular mechanics (MM)/generalized born surface area (GBSA) [73,74] following the described theory [75]. Additional molecular dynamics (MD) calculations were performed, aiming to confirm the predicted stability of the best-ranked drug-target system. Specifically, MD trajectories were generated with the graphic processing unit (GPU)-accelerated version of Desmond that successfully allows them to mimic interactions in a drug design [76]. The MD simulation is completed by producing trajectories of 100 ns. All calculations were conducted with Schrödinger (Schrödinger Release 2021-3).

### 2.2. Cell Culture

The Santiago Hospital Ethics Committee (CAEIG-2016/258) approved the informed consent, the signature for which is mandatory for all participant donors. Articular issue samples from healthy and OA patients were, respectively, obtained from dead and total knee replacement surgery donors [77]. Primary joint cells (chondrocytes, osteoblasts, and synoviocytes), isolated from their respective articular tissues, murine chondrogenic ATDC5 (RIKEN Cell Bank, Tsukuba, Japan), human osteoblastogenic SaOs2, and human synovial-sarcoma SW982 (ATCC, Manassas, VA, USA), were initially cell-cultured in P100 plates with DMEN F12 cell medium supplemented with fetal bovine serum (FBS) at 5% (mouse cells) or 10% (human cells), 2% Glutamine, and 2% penicillin-streptomycin, plus 0.2% sodium selenite and 0.1% transferrin (mouse cells) [22,23,77,78].

Only early cell passes (P) comprised of P_0_–P_3_ (primary cells) and P_5_–P_35_ (non-primary cells) were used to keep the cellular phenotype unaltered. Cells were seeded in cell plates (P12 or P100) and after 4 h, the medium was renewed with FBS deprived medium [22,23,77,78]. All cell culture reagents were purchased from Sigma-Aldrich (Sant Louis, MO, USA) except otherwise mentioned.

### 2.3. Cell Treatment

The concentration of use for lipopolysaccharide E. coli 026:B6 (LPS) (100 ng/mL), a pathogen-associated molecular pattern (PAMPs), and IL1β (0.1 ng/m) (Tebu-Bio, France) was selected from the literature [22] to be used, respectively, as a canonical agonist for TLR4 and IL1R complex. The specific TLR4 antagonist CLI-095/TAK-242 (1.00 µM) (InvivoGen, San Diego, CA, USA) was used as a TLR4 inhibitor [22,79]. We selected low concentrations of BBA (0.10–5.00 µM) (Merck, Darmstadt, Germany) to facilitate clinical transferability. All cell culture reagents were purchased from Sigma-Aldrich (Sant Louis, MO, USA) except otherwise mentioned. PubChem: IL1β CID 159483, CLI-095 CID 9919285, BBA CID 168928.

### 2.4. Metabolic and Biochemical Assays

The accumulation of nitrite (NO_2_^−^) levels was measured by Griess assay (Merck, Darmstadt, Germany) [22,77]. Nitrite measurement serves as an indirect determination of the innate immune response factor and ROS element, the nitric oxide (NO). Cell viability was determined by 3-(4,5-dimethylthiazol-2-yl)-2,5-diphenyltetrazolium bromide (MTT) assay (Merck, Darmstadt, Germany) [22,77]. Treatment concentrations were adjusted upon MTT and Griess assay dose-response results. Caspase 1 (CASP1) enzymatic activity was determined by Ac-Valine-Alanine-Asparagine-pNA (pNA) assay (Abbkine, Wuhan, China), and phosphoproteome was determined by Green Malachite assay (Merck, Darmstadt, Germany).

### 2.5. Transcriptomic Silencing

A specific short interference ribonucleic acid RNA (siRNA) was used to transiently silence the messenger RNA (mRNA) gene expression of NLRP3 (siNLRP3) and TLR4 (siTLR4) [80]. Briefly, cells were plated at a density of 50,000 cells/well (P24 plate), washed with Optimen and DMEN F12 (2% FBS) after 4h, and incubated for 1h with DMEN F12 (2% FBS). Then siRNA mix (15 nM) was administered for 24 h before pharmacological treatments such as LPS and IL1β. Both TLR4 and NLRP3 siRNA mix (15 nM) from TriFECTa DsiRNA kit (IDT, Coralville, IA, USA) contained 3 different oligos (sequence data not available) in equal proportion. Silencing effectiveness and specificity were validated in each experiment (*n* = 6) through positive (Hypoxanthine Guanine Phosphoribosyltransferase S1; HPRT-S1) and negative (scramble) controls. We confirmed the mRNA levels reduction of HPRT (71–92%) by positive controls, as well as TLR4 and NLRP3 (61–81%) by siTLR4 and siNLRP3 treatments. Furthermore, we confirmed the inability of negative controls to knock down NLRP3, TLR4, or HPRT mRNA levels.

### 2.6. Transcriptomic Profiling 

The gene expression (mRNA) of multiple genes (Supplementary data S1) was analyzed by comparative reverse transcription (RT)—quantitative polymerase chain reaction (qPCR) [22,77]. Briefly, the mRNA was isolated from cell cultures (2.5 × 10^5^ cells/well) using the trizol-chloroform column-based protocol of EZNA Total RNA Kit I (Omega Bio-Tek, Norcross, GA, USA) [77,81]. The mRNA was purified using deoxyribonucleic acid (DNA) enzyme DNAsa I, normalized and quantified by Nanodrop (260 nm). The mRNA (500 µg) was retrotranscribed to copy DNA (cDNA) using the High-Capacity RNA-To-cDNA Kit (Thermo Fisher Scientific, Waltham, MA, USA) [82]. The RT-qPCR determinations were performed under the minimum Information for publication of quantitative real-time PCR experiments (MIQE) guidelines [83]. Briefly, the RT-qPCRs were performed using cDNA, iTaq Universal SYBR Green Supermix (BioRad, Hercules, CA, USA), and KiCqStart SYBR Green primers (Merck, Darmstadt, Germany). These include TLR4, IL1R1, Heatshock Protein 90 αα1 (HSP90AA1), Mitogen-activated protein kinase (MAPK) 14 (MAPK14) or p38, CASP1, Nucleotide-binding oligomerization domain, NLRP3, IL6, IL1β, lipocalin 2 (LCN2), cyclooxygenase 1 (COX1), COX2, intercellular adhesion molecule 1 (ICAM1), vascular cell adhesion protein 1 (VCAM1), monocytes chemoattractant protein 1 (MCP1), and nicotinamide phosphoribosyl transferase (NAMPT), as well as catabolic factors such as metalloproteinase 1 (MMP1), -3 (MMP3), -9 (MMP9), -13 (MMP13), and A disintegrin and metalloproteinase with thrombospondin motifs 4 (ADAMTS4), aggrecan (ACAN), collagen 1 α1 (COL1A1), and collagen 2 α1 (COL2A1).

Furthermore, we used dependent or endogen control (Low-ROX dye) and the independent reference control (hypoxanthine phosphoribosyl transferase 1; HPRT1). The RT-qPCRs were performed under a FAST thermal profile (95 °C for 20″; 95 °C for 1″ and 60 °C for 20″ during 45 cycles) in a QuantStudio3 (QS3) thermocycler (Thermo Fisher Scientific, Waltham, MA, USA) [84,85]. 

### 2.7. Proteome Profiling

Protein levels were analyzed by enzyme-linked immunosorbent assay (ELISA) and matrix-assisted laser desorption/ionization (MALDI)—time of flight (TOF). Briefly, proteins were isolated from cell cultures using RIPA 1X buffer (Merck, Darmstadt, Germany) with HALT protease and phosphatase inhibitors (Thermo Fisher Scientific, Waltham, MA, USA) and quantified using the bovine serum albumin (BSA) Pierce Bradford assay (ThermoFisher, USA).

The cellular and secreted (supernatant) levels of IL6 and IL1β proteins were determined in joint cells by mouse and human ELISA antibodies (Abbkine, Wuhan, China) and quantified (450 nm and 570 nm subtraction) in a Multiskan SkyHigh plate reader (Thermo Fisher Scientific, Waltham, MA, USA). Similarly, cellular proteome and secretome were determined in mouse and human chondrocytes by qualitative shotgun data-dependent acquisition (DDA) MALDI-TOF method [86,87,88,89]. The DDA was performed in a micro-liquid chromatography (mLC) mass spectrometry (MS)/MS attached to a hybrid quadrupole-TOF/triple-TOF 6600 and output data was filtered by 5% false discovery rate (FDR) and *p* ≤ 0.05 [89]. The ortholog migration algorithm from REACTOME conversed mouse and human proteomes [90]. The Venn diagram and interactome from FunRich software analyzed protein profiles [91]. The molecular function and pathway enrichment from REACTOME identified different medical and biological associations [90].

### 2.8. Statistical Analysis and Sample Size

The sample size (*n*) of the whole study was established in 6 independent biological replicas from different patients or cell passes and 3 dependent technical replicas. The availability of non-primary cells of early pass was unlimited. On the contrary, the availability of primary cells isolated from OA patients or healthy corpses was very limited. Patients were excluded from the study (CAEIG-2016/258) if they were diagnosed with microcrystalline arthritis or any other infectious process. Moreover, even if they were not diagnosed, a further analysis of the fresh sample was also needed. Similarly, the presence of any other pathology apart from OA directly excluded those samples. All included samples presented sufficient cartilage and bone to perform the cell isolation. Nevertheless, only 3 healthy samples and 3 OA samples presented sufficient synovium to be removed and processed to isolate synoviocytes. Biochemical assays (Griess and MTT) required 10^4^ cells (P96 plate), transcriptomics (RT-qPCRs) required 10^5^ cells (P12), metabolic assays required 3 × 10^5^ cells (P6) and proteomics (ELISA and MALDI-TOF) required 3.5 × 10^6^ cells (P100) per data point. Overall, the use of primary cells was optimized to acquire the largest amount of data points. MALDI-TOF proteomics analysis was the most cell-demanding technique and, considering that thousands of technical replicas runs are performed, we optimized its sample size to 3 independent biological replicas.

All datasets were expressed as the mean ± standard error mean (SEM) and normalized by the “Control” treatment data. Gene expression (mRNA) data was obtained by the RT-qPCR threshold cycle (ΔΔCt), also known as the ΔΔ quantification cycle (ΔΔCq). Gene expression was normalized by reference gene HPRT1 and analyzed by comparative transcriptomics. Fold-change (log_2_) units were used in the proteome heatmaps or column charts.

The whole manuscript used *The New England Journal of Medicine* (NEJM)’s statistical significance method of representation. Specifically, a *p*-value of: <0.001 (***), <0.002 (**), <0.033 (*), and <0.12 (blank space or ns). Data distribution (Shapiro–Wilk and Kolmogorov–Smirnov normality tests) determined the use of parametric or non-parametric tests. Data from primary human cells were analyzed by non-parametric Kruskal–Wallis or Mann–Whitney test if needed. Otherwise, data were analyzed by parametric One Way-ANOVA and Tukey post-test or unpaired *t*-test Welch’s correction if needed.

## 3. Results

### 3.1. Determining the Proteome Profile of TLR4/IL1R-Stimulated Primary Human OA Chondrocytes

Anti-OA properties have been associated with BSE mix through NFκB pathway inhibition; nonetheless, the results were not consistent enough. Consequently, no international organizations such as the Food and Drug Administration (FDA) or the European Medicines Agency (EMA) have approved BSE mix or any individual component as a drug for clinical use. As such, our study focused on three pillars: the BSE mix single element BBA, the use of a primary joint cell model, and the implementation of novel techniques such as computational chemistry.

**Figure 1:**
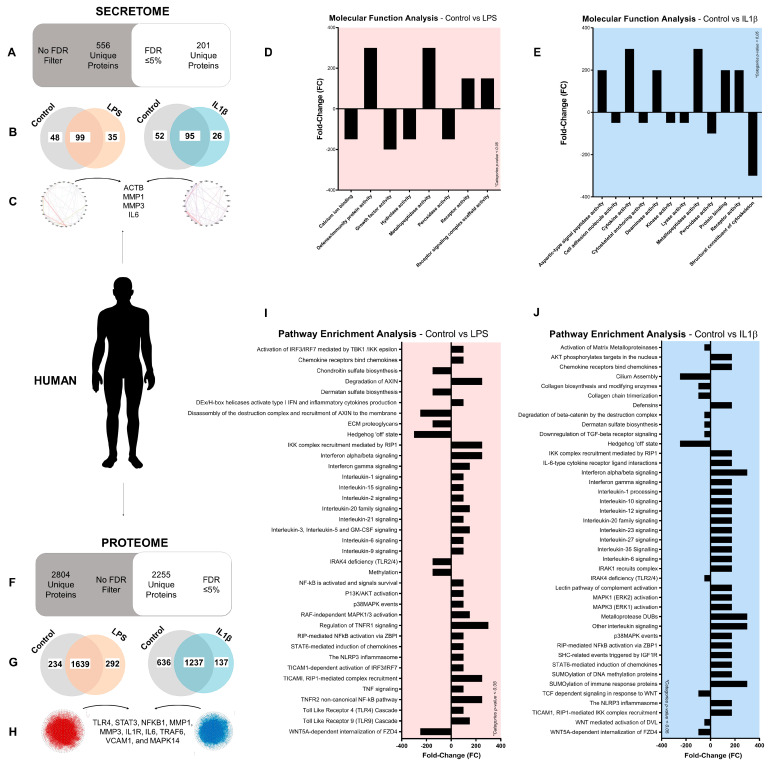
Determining the human secretome and proteome of TLR4/IL1R-activated OA chondrocytes: (**A**–**J**) Secretome and proteome profile by DDA MALDI-TOFF of human OA chondrocytes stimulated with LPS (100 ng/mL) or IL1β (0.1 ng/mL) for 48 h. Independent biological replicas (*n* = 3) were normalized (control), expressed as the mean and /or fold-change (FC); (**A**) human secretome profile of non-filtered (FDR ≤ 0% = 556) and filtered (FDR ≤ 5% = 201) secreted proteins; (**B**) Venn diagram (FDR ≤ 5%) of TLR4 (LPS)/IL1R (IL1β)-activated and non-activated (Control) human OA chondrocytes secretome; (**C**) interactome plot (FDR ≤ 5%) of TLR4/IL1R-activated human OA chondrocytes secretome; (**D**,**E**) enrichment analysis of molecular functions (FDR ≤ 5%; fold-change) in the TLR4/IL1R-activated human OA chondrocytes secretome; (**F**) human proteome profile of non-filtered (FDR ≤ 0% = 2804) and filtered (FDR ≤ 5% = 2255) cellular proteins; (**G**) Venn diagram (FDR ≤ 5%) of TLR4 (LPS)/IL1R (IL1β)-activated and non-activated (Control) human OA chondrocytes proteome; (**H**) interactome plot (FDR ≤ 5%) of TLR4/IL1R-activated human OA chondrocytes proteome; (**I**,**J**) pathway enrichment analysis (FDR ≤ 5%; fold-change) ofTLR4/IL1R-activated human OA chondrocytes proteome.

Potential BBA targets described in the literature are downstream of the TLR4/IL1R signaling pathway, including NFκB. In this sense, we analyzed the secretome and proteome of TLR4/IL1R-activated human OA chondrocytes (hOC) isolated from OA patients to validate novel and already described inhibitory targets (Figure 1).

The secretome profile (FDR ≤ 5%) of hOC stimulated for 48 h with the TLR4 agonist LPS (100 ng/mL) and IL1R agonist IL1β (0.1 ng/mL]) displayed 201 unique proteins (Figure 1A). Multiple proteins were consistently expressed, whereas others were modulated after TLR4 (down 48/up 35 proteins) and IL1R (down 52/up 26 proteins) activation (Figure 1B). Interestingly, the interactome analysis showed that the most critical and highly associated proteins in TLR4/IL1R-activated hOC were β-actin (ACTB), MMP1, MMP3, and IL6 among others (Figure 1C). Furthermore, enrichment analysis of molecular functions in TLR4 and IL1R-activated hOC identified multiple categories associated with anabolism depleted whereas the ones associated with catabolism were enriched (Figure 1D,E).

The human proteome profile (FDR ≤ 5%) displayed 2255 unique cellular proteins (Figure 1F). Among them, multiple proteins were associated with TLR4 (down 234/up 292 proteins)/IL1R (down 636/up 137 proteins) activation (Figure 1G). At the centermost of the TLR4/IL1R-activated interactome plot, TLR4, Signal Transducer and Activator Transcription 3 (STAT3), NFKB1, MMP1, MMP3, IL1R, IL6, Tumor Necrosis Factor Receptor Associated Factor 6 (TRAF6), VCAM1, and MAPK14 proteins were found among others (Figure 1H). The similarity of results between TLR4 and IL1R-activated human proteomes was further observed in the pathway enrichment analysis of REACTOME innate immunity (Figure 1I,J). Interestingly, TLR4/IL1R downstream signaling pathways MAPK/p38, NFκB, tumor necrosis factor (TNF), Interferon αβ (IFNαβ), ROS, and NLRP3 were found enriched (Figure 1I,J). Contrarily, we found depleted extracellular matrix (ECM) synthesis and maintenance pathways (Figure 1I,J).

The use of animal or cellular mouse models is essential in translational medicine research as an initial step due to the scarcity of primary human OA experimentation. In this sense, we replicated the cellular proteome analysis workflow in TLR4/IL1R-activated mouse ATDC5 chondrocytes (Figure S1A–D). Interestingly, the mouse proteome profile identified a similar pattern of induced and inhibited proteins to the human proteome profile (Figure S1A,B). Moreover, both TLR4 and IL1R-activated mouse ATDC5 chondrocytes put at the centermost of the interactome (Figure S1C) the pleiotropic Polyubiquitin C (Ubc), a protein known to promote NFκB pathway activation [92]. In line with this, categories associated with MAPK/p38, NFκB, and ROS were found enriched among others, whereas ECM pathways were found depleted (Figure S1D,E).

### 3.2. Predicting the Binding Affinity of β Boswellic Acid to Potential Proteome Profile Targets

**Figure 2:**
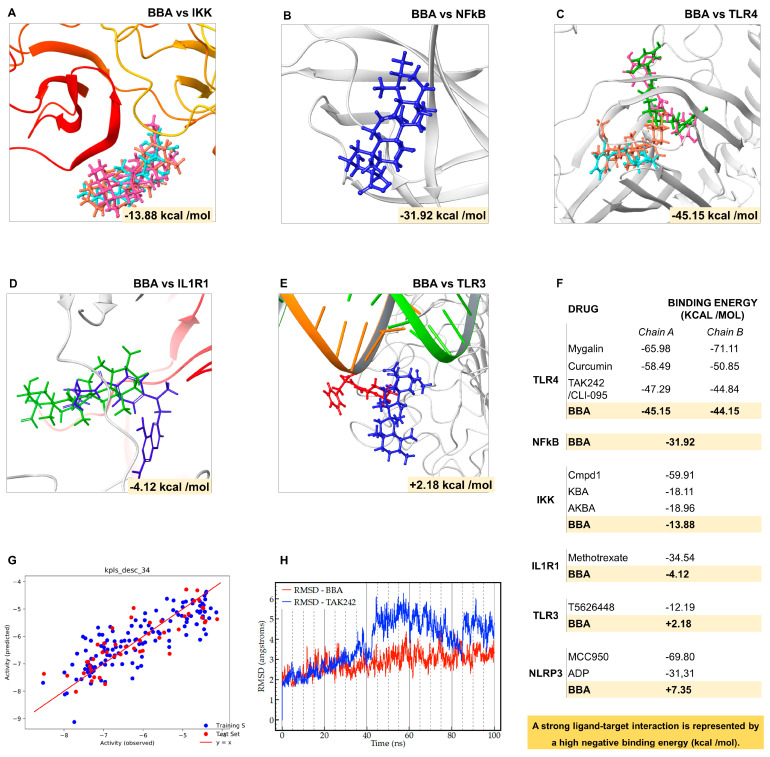
Predicting the binding affinity of β boswellic acid to biological targets: (**A**–**F**) The ability of ligands (BBA, Cmpd1, KBA, AKBA, Mygalin, Curcumin, TAK242/CLI-095, Methotrexate, T5626448, MCC950, and ADP) to dock targets (**A**) IKK, (**B**) NFkB, (**C**) TLR4, (**D**) IL1R1, (**E**) TLR3, and (**F**) NLRP3 was ranked (**F**) according to the predicted binding energy and displayed the best result (pose) in each analysis. The more negative energy, the stronger the interaction ligand-target is. The selected computational order ranks relative to the affinity; (**G**) BBA cell permeability (red dots) using a quantitative structure–activity relationships (QSAR) model trained with cell permeability data of 190 compounds (blue dots) and obtaining a log P_e_ = 5.38; (**H**) molecular dynamics (MD) trajectories (100 ns) and root means-square deviation (RMSD) were used to measure the structural dynamic stability of a ligand docked in a target binding cite.

Secretome and proteome pointed to NFκB, MAPK/p38 (MAPK14), TLR4, IL1R, and NLRP3 as the most promising targets for BBA inhibitory properties. The first three develop their activity in the cytosol; therefore, BBA needs to cross the cell membrane to perform inhibitory effects, whereas the others are innate immune receptors bound to a membrane. In this sense, through computational chemistry we explored the binding affinity of β boswellic acid (BBA) towards these biological targets (Figure 2).

The development of NFκB signaling inhibitors such as the Cmpd1 inhibitor uses the IKK as a prime target [66]. This inhibitor binds to the IKKβ at the hinge loop connecting the N and C lobes, a region that recognizes the adenine within the adenosine triphosphate (ATP) [66]. Our simulations (Figure 2A,F) revealed that Cmpd1 is located at that region with significant large energy (−59.91 kcal/mol). On the contrary, 11-keto-β-boswellic acid (KBA, −18.11 kcal/mol), acetyl-11-keto-β-boswellic acid (AKBA, −18.96 kcal/mol), or BBA (−13.88 kcal/mol) are anchored with less negative binding energy; that is, with a less intense interaction (Figure 2A,F). Consequently, these data demonstrated that the observed activity for BBA and derivatives was not associated with the IKK-related NFκB inhibition mechanism. The unsuccessful interaction results motivated us to assess the interaction of BBA against NFκB using the Müller et al. NFκB p50 (1SVC) [93] homodimer bound to DNA. We explored the whole protein surface, and the best-ranked poses were retained for MM/GBSA refinement. Interestingly, the simulations suggested that BBA binds NFκB with a strong interaction energy of −31.92 kcal/mol (Figure 2B,F).

This successful result led us to study targets upstream of NFκB such as the human TLR4 complex. This complex is formed by two subunits of TLR4 protein (Chain A and B) and two subunits of accessory molecule MD2 [94] (Figure 2C,F). TLR4 is a promiscuous innate immune response (IIR) receptor capable of binding to a large diversity of host (DAMPs) and non-host (PAMPs) derived structures. Our molecular model for TLR4 was designed using the crystal deposited in the PDB with code 3VQ1 [95] (Figure 2C,F) Interestingly, the binding affinity (Figure 1A) of BBA towards the human TLR4 complex (−45.15 Kcal/mol for Chain A) was found energetically on par with the specific pharmacological TLR4 inhibitor CLI-095/TAK242 (−47.29 Kcal/mol). Furthermore, similar results were obtained for curcumin (−58.49 Kcal/mol) and mygalin (−65.98 Kcal/mol), two other candidates as TLR4 inhibitors for clinical practice (Figure 2A,F). Moreover, a similar trend was observed for TLR4 complex Chain B (Figure 2A,F).

Considering that computational analysis ranked BBA-TLR4 as the most promising target for BBA, we decided to further assess two TLR4-related receptors namely the IL1R and TLR3 complexes (Figure 2D–F). The binding affinity of IL1R-BBA (−4.12 Kcal/mol) was found lower than IL1R-Methotrexate (−34.54 Kcal/mol), the main IL1R inhibitor (Figure 2D,F). The TLR3 shares with the TLR4 part of the signaling cascade but it is activated by dsDNA and inactivated by inhibitors such as T5626448 (−12.19 Kcal/mol); nevertheless, the binding affinity TLR3-BBA (+2.18 Kcal/mol) was found inexistent (Figure 2E,F). Finally, we addressed the potential affinity NLRP3-BBA, a signaling pathway downstream to TLR4 and IL1R. We compared the binding energy of the best docking pose predicted for BBA against the NLRP3 agonist ADP and antagonist MCC950 [96]. According to the positive binding energy shown in our simulations, BBA (+7.35 kcal/mol) does not fit into that position, while both MCC950 (−69.80 kcal/mol) and ADP (−31.31 kcal/mol) efficiently dock to the binding site (Figure 2F).

These accumulated results evidenced a predilection of BBA to bind the receptor TLR4; nonetheless, they do not exclude the possibility of directly interacting with NFκB after crossing the cellular membrane. We used a 190-compound dataset to train [97] (training blue points) the Quantitative Structure Activity Relationships (QSAR) [98] software to predict the effective drug cell permeability coefficient (log P_e_). Indeed, trained QSAR predicted (testing red points) an effective permeability coefficient of BBA (log P_e_ = 5.38) that matches the naloxone permeability, a drug known to cross the cell membrane but also capable of binding and inhibiting the TLR4 signaling [23] (Figure 2G).

This potential dual dynamic is interesting and not yet documented by the literature; onwards, we focused on the BBA binding effect on the TLR4 complex. In fact, we performed molecular dynamics (MD) simulations and root means-square deviation (RMSD) monitoring to test BBA-TLR4 docking strength against the CLI-095-TLR4 complex (Figure 2H). Specifically, RMSD values reveal that BBA (red line) is stabilized with an RMSD variation of 3 Å after 20 ns, whereas the reference inhibitor TAK242 required a larger RMSD window of 5 Å after 40 ns (Figure 2H). The smaller RMSD variation associated with BBA confirms the stability of the complex under biological conditions. Consequently, MD simulations ensured dynamic binding stability (100 ns trajectory) and RMSD monitoring ensured structural stability through a key TLR4 residue (Arg90) comparable for both BBA-TLR4 and CLI-095-TLR4 (Figure 2H).

Altogether, computational pharmacology found the TLR4 complex as the most probable target with large binding interaction energy comparable to the value predicted for one of the golden standard inhibitors, namely, CLI-095 or TAK242.

### 3.3. BBA Effects on Cell Viability and ROS Secretion in Chondrocytes

Computational docking predicted a higher binding affinity of BBA towards the human TLR4 complex. This BBA feature was compatible with TLR4 antagonism. Moreover, BBA was predicted not to bind the IL1R complex. Previous studies reported that TLR4 blockade inhibited both TLR4 and IL1R signaling [22]. Thus, we validated the in silico results of BBA actions in an in vitro model to determine its potential modulation of the TLR4/IL1R signaling cascade.

**Figure 3:**
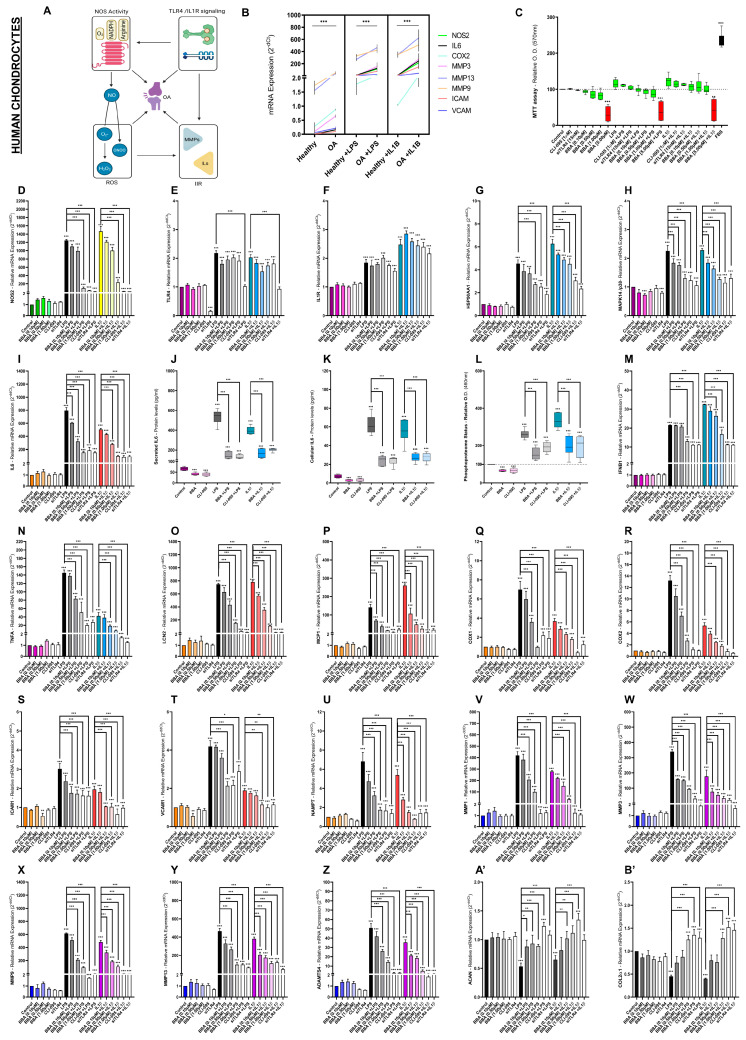
BBA effects on TLR4/IL1R-mediated innate immune response in primary human OA and healthy chondrocytes: (**A**) diagram of the interconnection between Osteoarthritis (OA) and TLR4/IL1R signaling, NOS activity, Reactive Oxygen Species (ROS) as well as Innate Immune Responses (IIR); (**B**–**B’**) primary human healthy (**B**) or OA (**B**–**B’**) chondrocytes (hOC) were treated with BBA (0.10–5 µM), CLI-095 (1 µM), or siTLR4 (15 nM) for 24 h and cotreated with LPS (100 ng/mL) or IL1β (0.1 ng/mL) for another 48 h. Independent biological replicas (*n* = 6) were normalized (control), expressed as the mean ±SEM and represented by the statistical significance NEJM system as *** (*p*-value < 0.001), ** (*p*-value < 0.002), * (*p*-value < 0.033); (**C**) MTT cell viability assay in hOC chondrocytes displaying cell inviable doses (red), cell-viable doses (green) and FBS as a positive control; (**B**,**D**–**I**,**M**–**B’**) RT-qPCR gene expression (mRNA) of NOS2, TLR4, IL1R1, HSP90AA1, MAPK14/p38, LCN2, COX1, COX2, ICAM1, VCAM1, MCP1, NAMPT, MMP-1, -3, -9, -13, ADAMTS4, and/or ACAN and COL2α1 in primary human healthy or OA chondrocytes; (**J**–**K**) ELISA of secreted (**J**) or cellular (**K**) IL6 protein (pg/mL) in primary hOC; (**L**) Green malachite assay (Relative O.D. 460 nm) of phosphoproteome status in primary hOC.

Among the different pathways associated with TLR4/IL1R signaling, ROS generation was found highly enriched in the human and mice proteome profiles (Figure 2I,J and Figure S1D,E). Specifically, NO generation was a common denominator between TLR4 and IL1R activation (Figure 2I,J and Figure S1D,E). In fact, NO has been associated with the development of OA, the promotion of innate immune responses, the increase of articular catabolism, and the generation of more ROS (Figure 3A) [99,100,101]. Before proceeding to determine the effects of potential therapeutic BBA (0.10–5 µM) doses on ROS generation, we compared the basal innate immune response tone (mRNA) between primary human chondrocytes isolated from OA patients or healthy corpses (Figure 3B). Primary human OA chondrocytes (hOC) presented higher mRNA levels for all the studied IIR factors (Figure 3B). Furthermore, OA joint cells were found more sensitive to TLR4/IL1R activation than healthy ones (Figure 3B). Next, we evaluated the balance between cell viability, the accumulation of NO subproduct NO_2-_, and NOS2 gene expression (mRNA) to determine the effects of potential therapeutic BBA (0.10–5 µM) doses on ROS generation in primary hOC (Figure 3C,D) as well as in mice ATDC5 chondrocytes (Figure S1F–I).

After LPS (100 ng/mL) and IL1β (0.1 ng/mL) stimulation, cell viability was unaltered (Figure 3B,C and Figure S1F,G) and accumulation of NO_2_^−^ was increased (Figure S1H,I). Interestingly, unlike high concentrations of BBA [5 µM], cotreatment with BBA (0.10–1 µM) for 48 h decreased NO_2-_ accumulation in a dose-response manner without altering cell viability (Figure 3B,C and Figure S1F–I). Next, we evaluated BBA (0.10–1 µM) effects on NOS2 gene expression, also known as inducible nitric oxide synthase (iNOS). Results were consistent with ROS-NO_2_ determination; activation of TLR4/IL1R increased NOS2 mRNA levels and BBA (0.10–1 µM) decreased them in a dose-response manner (Figure 3D and Figure S1J).

Considering these results, we evaluated whether BBA effects were bound to the modulation of the expression of TLR4 or IL1R mRNA levels. Gene expression of TLR4 and IL1R showed an increase in mRNA levels after LPS/IL1β stimulation but no modulation after BBA co-treatment (Figure 3E,F and Figure S1K,L).

Interestingly, treatment with the specific TLR4 small-molecule inhibitor CLI-095 (1 µM) or a siRNA anti-TLR4 (siTLR4) (15 nM) recapitulated BBA treatment effects (Figure 3C–F and Figure S1J–L). Furthermore, siTLR4 silencing treatment severely downregulated TLR4 mRNA levels but did not alter IL1R mRNA levels (Figure 3E,F and Figure S1K,L). Moreover, the blockade of the TLR4 complex by CLI-095 treatment did not alter TLR4 nor IL1R mRNA levels (Figure 3E,F and Figure S1K,L).

### 3.4. BBA Effects on TLR4/IL1R-Mediated IIRs in Primary Human OA Chondrocytes

Once the inhibitory effects of BBA in the NOS2 axis were demonstrated, we further explored other TLR4/IL1R-modulated pathways found enriched in the proteome analysis including MAPK p38/NFκB, IFNαβ, and TNF (Figure 1; Supplementary data S2). The MyD88-dependent MAPK p38/NFκB downstream pathway is central to TLR4/IL1R signaling. We evaluated the mRNA expression of common specific elements in the MAPK p38 and NFκB pathway, namely, HSP90AA1, MAPK14 or p38, and IL6 in primary human OA chondrocytes (hOC). We found increased mRNA levels of HSP90AA1, p38, and IL6 after TLR4/IL1R activation (Figure 3G–I) and decreased ones after cotreatment with BBA (0.10–1 µM), CLI-095 (1 µM), and siTLR4 (1 µM) in a dose-response dynamic (Figure 3G–I). To validate these data, we further tested the intracellular and secreted levels of IL6 in these cells. The TLR4/IL1R activation increased secreted (Figure 3J) and cellular (Figure 3K) protein concentration of IL6 (up to 584 pg/mL) while BBA and CLI-095 cotreatment inhibited it. Consistent with a decreased activation of MAPK p38/NFκB pathway by BBA, we observed in TLR4/IL1R-activated hOC a reduction in the total phosphoproteome after BBA treatment (Figure 3L). Interestingly, cotreatment with BBA or CLI-095 also reduced the protein concentration of IL6 and phosphoproteome levels in non-stimulated cells (Figure 3J–L).

Next, we evaluated the TNF pathway element TNFα, the MyD88-independent IFNαβ pathway element IFNβ1, and highly studied innate immune response factors associated with the TLR4/IL1R pathway including MAPK p38/NFκB downstream signaling factors in the OA context. Remarkably, most of these factors were directly identified in the secretome and proteome profiles (Figure 1A–J and Figure S1A–E). At the transcriptomic level, inflammatory factors (IFNβ1, TNFα, LCN2, COX1, COX2, ICAM1, VCAM1, MCP1, and NAMPT) and catabolic factors (MMP1, MMP3, MMP9, MMP13, and ADAMTS4) mRNA levels were increased after LPS and IL1β stimulation of primary hOC (Figure 3M–Z). In contrast, mRNA levels of anabolic factors (ACAN and COL2A1) were decreased after LPS and IL1β stimulation of primary hOC (Figure 3A’–B’). Gene expression (mRNA) of inflammatory and catabolic factors decreased in a dose-response manner after cotreatment with BBA, CLI-095, or siTLR4 (Figure 3M–Z). In contrast, gene expression (mRNA) of anabolic factors was increased and reverted to basal control levels after BBA, CLI-095, or siTLR4 cotreatment (Figure 3A’–B’). Similar results were obtained in mice ATDC5 chondrocytes (Figure S1M,N and Figure S2A–O).

### 3.5. BBA Effects on TLR4/IL1R-Mediated IIRs in Primary Human OA Osteoblasts

**Figure 4:**
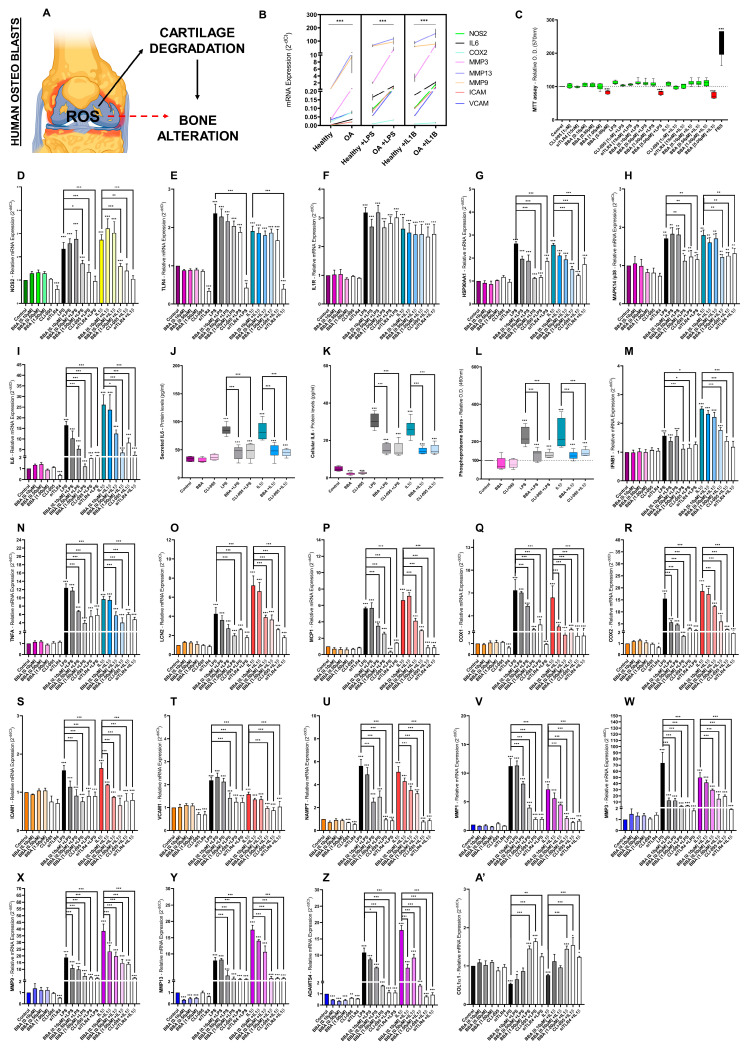
BBA effects on TLR4/IL1R-mediated innate immune response in primary human OA and healthy osteoblasts: (**A**) diagram of the interconnection between articular tissues in osteoarthritis; (**B**–**A’**) primary human healthy (**B**) or OA (**B**–**A’**) osteoblast (hOB) were treated with BBA (0.10–5 µM), CLI-095 (1 µM) or siTLR4 (15 nM) for 24 h and cotreated with LPS (100 ng/mL) or IL1β (0.1 ng/mL) for another 48 h. Independent biological replicas (*n* = 6) were normalized (control), expressed as the mean ± SEM and represented by the statistical significance NEJM system as *** (*p*-value < 0.001), ** (*p*-value < 0.002), * (*p*-value < 0.033); (**C**) MTT cell viability assay in hOB osteoblasts displaying cell inviable doses (red), cell-viable doses (green) and FBS as a positive control; (**B**,**D**–**I**,**M**–**A’**) RT-qPCR gene expression (mRNA) of NOS2, TLR4, IL1R1, HSP90AA1, MAPK14/p38, LCN2, COX1, COX2, ICAM1, VCAM1, MCP1, NAMPT, MMP-1, -3, -9, -13, ADAMTS4, and/or COL1A1 in primary human healthy or OA osteoblasts; (**J**,**K**) ELISA of secreted (**J**) or cellular (**K**) IL6 protein (pg/mL) in primary hOB; (**L**) Green malachite assay (Relative O.D. 460 nm) of phosphoproteome status in primary hOB.

The OA disease progression begins degrading the cartilage and eventually alters the bone structure (Figure 4A). As such, we evaluated the BBA inhibitory effects on the TLR4/IL1R downstream pathways in primary human OA osteoblasts (hOB) isolated from OA patients. Remarkably, primary hOB presented higher mRNA levels for all the studied IIR factors. Similarly, hOB was more sensitive to TLR4/IL1R activation than healthy ones (Figure 4B). In this sense, we used BBA (0.10–1 µM), CLI-095 (1 µM), and siTLR4 (1 µM) doses that did not alter primary hOB cell viability (Figure 4B–A’).

Interestingly, ROS/NOS2 and MAPK p38/NFκB pathway (Figure 4D–L) presented a similar transcriptomic profile to hOC chondrocytes (Figure 3D–L). The 48-h stimulation with LPS and IL1β increased NOS2, TLR4, IL1R, HSP90α, p38, and IL6 mRNA levels, as well as IL6 protein levels and the phosphoproteome status (Figure 4D–L). In line with hOC results, BBA, CLI-095, and siTLR4 severely reduced the expression levels of all the studied factors except IL1R (Figure 4D–L). Besides siTLR4, neither BBA nor CLI-095 inhibited TLR4 mRNA levels (Figure 4E).

The transcriptomic profile of the other TLR4/IL1R downstream signaling factors including TNF and IFNαβ pathways in primary hOB osteoblasts was similar to the hOC chondrocytes. As such, stimulation with LPS and IL1β in primary hOB increased inflammatory factors (IFNβ1, TNFα, LCN2, COX1, COX2, ICAM1, VCAM1, MCP1, and NAMPT) and catabolic factors (MMP1, MMP3, MMP9, MMP13, and ADAMTS4) mRNA levels but decreased anabolic factor (COL1A1) mRNA levels (Figure 4M–A’). Consequently, BBA, CLI-095, or siTLR4 cotreatment increased anabolic factors to basal levels and decreased inflammatory and catabolic factors gene expression in a dose-response manner (Figure 4M–A’). Overall, the intensity of TLR4/IL1R-activation on hOB osteoblasts mRNA and protein levels was lower compared to hOC chondrocytes; nevertheless, BBA, CLI-095, or siTLR4 co-treatment effects were the same (Figure 3 and Figure 4).

### 3.6. BBA Effects on TLR4/IL1R-Mediated IIRs in Human OA Synoviocytes

**Figure 5:**
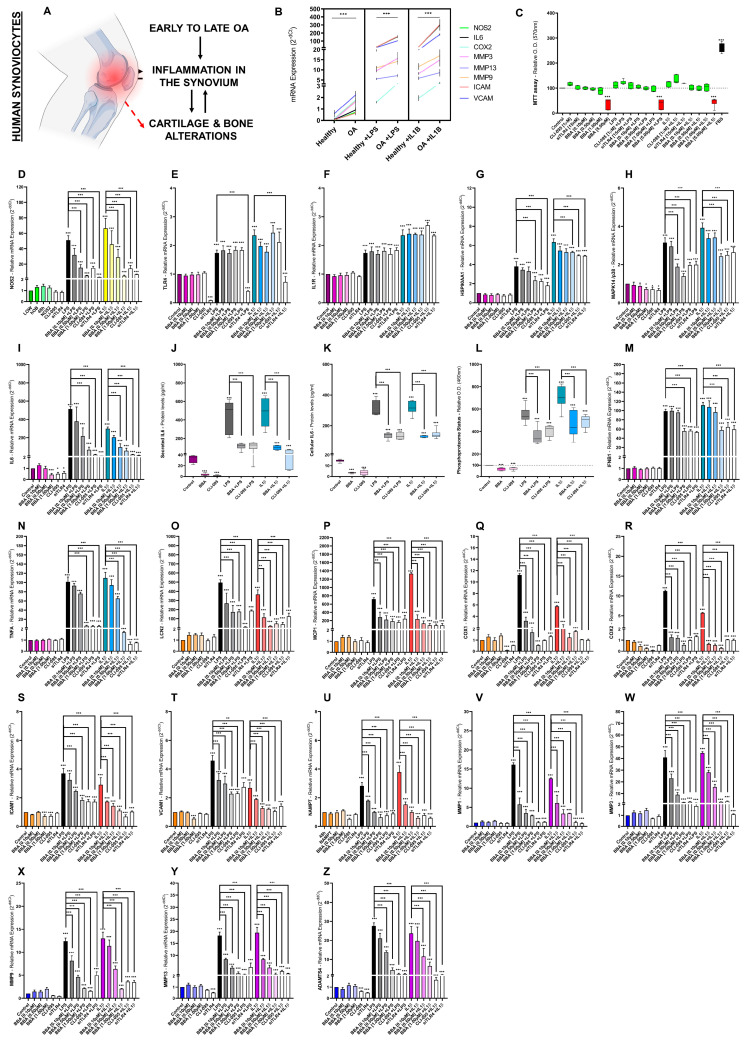
BBA effects on TLR4/IL1R-mediated innate immune response in human synoviocytes: (**A**) Diagram of the interconnection between articular tissues in osteoarthritis; (**B**–**Z**) primary human healthy (**B**), OA (**B**) and non-primary human SW982 (**C**–**Z**) synoviocytes (hSW) were treated with BBA (0.10–5 µM), CLI-095 (1 µM), or siTLR4 (15 nM) for 24 h and cotreated with LPS (100 ng/mL) or IL1β (0.1 ng/mL) for another 48 h. Independent biological replicas (*n* = 6) were normalized (control), expressed as the mean ±SEM and represented by the statistical significance NEJM system as *** (*p*-value < 0.001), ** (*p*-value < 0.002), * (*p*-value < 0.033); (**C**) MTT cell viability assay in hSW synoviocytes displaying cell inviable doses (red), cell-viable doses (green) and FBS as a positive control; (**B**,**D**–**I**,**M**–**Z**) RT-qPCR gene expression (mRNA) of NOS2, TLR4, IL1R1, HSP90AA1, MAPK14/p38, LCN2, COX1, COX2, ICAM1, VCAM1, MCP1, NAMPT, MMP-1, -3, -9, -13, and ADAMTS4 in human healthy (**B**), OA (**B**) and hSW synoviocytes (**D**–**I**,**M**–**Z**); (**J**,**K**) ELISA of secreted (**J**) or cellular (**K**) IL6 protein (pg/mL) in hSW synoviocytes; (**L**) Green malachite assay (Relative O.D. 460 nm) of phosphoproteome status in hSW synoviocytes.

The pathophysiology from early-to-late OA regularly causes inflammation of the synovium; in turn, inflamed synovia promotes the alteration of other joint tissues (Figure 5A). Consequently, we evaluated the BBA inhibitory effects in human synoviocytes. In this sense, primary human OA synoviocytes (hOS) presented higher mRNA levels for all TLR4/IL1R-associated innate immune factors (Figure 5B). In fact, hOS were more sensitive to TLR4/IL1R activation than healthy ones (Figure 5B). Nevertheless, the scarcity of primary human OA synoviocytes forced us to switch to human SW982 synoviocytes (hSW). Notably, the evaluation of BBA inhibitory effects on the TLR4/IL1R downstream pathways did not use doses of BBA (0.10–1 µM), CLI-095 (1 µM), and siTLR4 (1 µM) that altered hSW cell viability (Figure 5C–Z).

The activation of TLR4/IL1R for 48 h in hSW synoviocytes modulated the expression of all TLR4/IL1R-associated genes being studied (Figure 5D–Z). The innate immune factors (NOS2, TLR4, IL1R, HSP90α, p38, IL6, IFNβ1, TNFα, LCN2, COX1, COX2, ICAM1, VCAM1, MCP1, NAMPT, MMP1, MMP3, MMP9, MMP13, and ADAMTS4) were upregulated (mRNA). Similarly, TLR4/IL1R-activated hSW synoviocytes increased protein levels of secreted and cellular IL6 as well as the phosphoproteome status (Figure 5J–L).

The use of BBA, CLI-095, and siTLR4 downregulated (mRNA, protein, and phosphoproteome status) all factors except IL1R (Figure 5D–Z). Likewise, neither BBA nor CLI-095 inhibited TLR4 mRNA levels, only the siTLR4 was capable (Figure 5D–Z). Overall, the intensity of TLR4/IL1R-activation on hSW synoviocytes mRNA and protein levels was higher compared to hOC chondrocytes and hOB osteoblasts. Nonetheless, BBA, CLI-095, or siTLR4 co-treatment effects were very similar in the three human joint cells: chondrocytes (Figure 3), osteoblasts (Figure 4), and synoviocytes (Figure 5).

### 3.7. BBA Effects in Joint Cells on the NLRP3 Inflammasome Pathway

The proteome enrichment analysis of TLR4/IL1R-activated primary human OA chondrocytes identified specific pathways downstream of these receptors but upstream of relevant key factors such as MAPK p38 or NFκB. The transcriptomic and protein analysis further supported that these pathways could contribute to the mechanism of action of BBA inhibitory effects. Remarkably, the proteome profile identified the NLRP3 inflammasome signaling as a pathway enriched after the activation of TLR4 and IL1R (Figure 1 and Figure S1). Furthermore, the activation of the NLRP3 pathway triggers CASP1 that catalyzes the transformation of pro-IL1β into mature IL1β, the canonical agonist of IL1R1.

**Figure 6:**
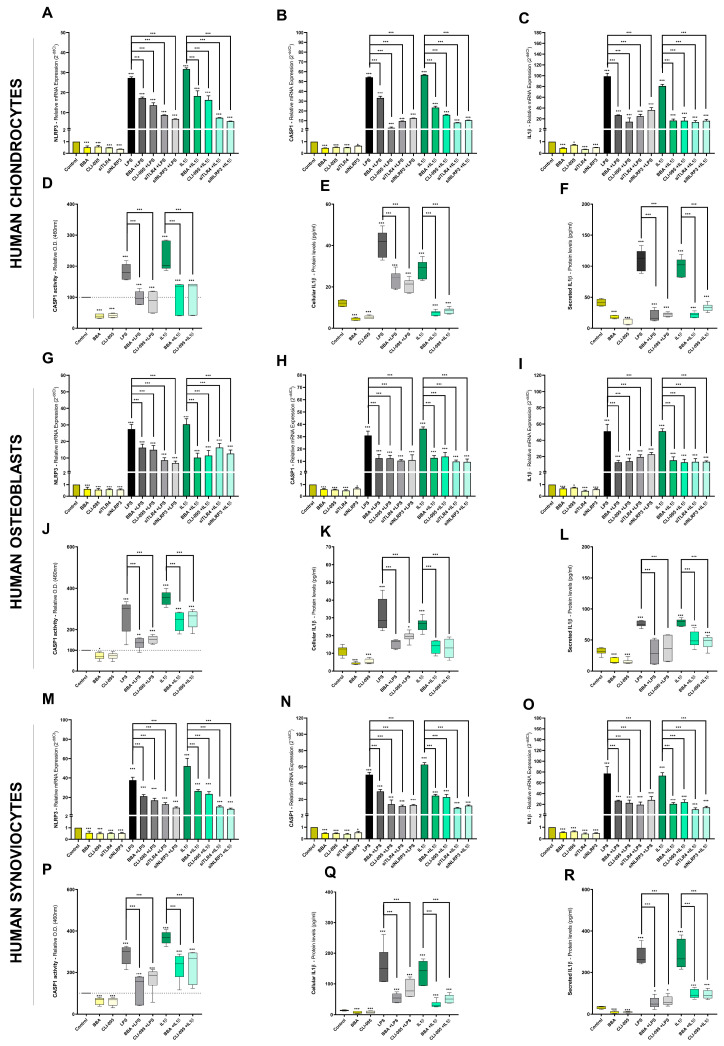
BBA effects on NLRP3-mediated innate immune response in primary joint cells: (**A**–**R**) Primary human OA chondrocytes (hOC), primary human OA osteoblasts (hOB), and human SW982 synoviocytes (hSW) were treated with BBA (1 µM), CLI-095 (1 µM), siTLR4 (15 nM) and siNRLP3 (15 nM) for 24 h and cotreated with LPS (100 ng/mL) or IL1β (0.1 ng/mL) for another 48 h. Independent biological replicas (*n* = 6) were normalized (control), expressed as the mean ± SEM, and represented by the statistical significance NEJM system as *** (*p*-value < 0.001), ** (*p*-value < 0.002), * (*p*-value < 0.033); (**A**–**C**,**G**–**I**,**M**–**O**) RT-qPCR gene expression (mRNA) of NLRP3, CASP1, and IL1β in hOC chondrocytes (**A**–**C**), hOB osteoblasts (**G**–**I**), and hSW synoviocytes (**M**–**O**); (**D**,**J**,**P**): CASP1 activity (pNA reaction) assay in hOC chondrocytes (**D**), hOB osteoblasts (**J**), and hSW synoviocytes (**P**); (**E**,**F**,**K**,**L**,**Q**,**R**) ELISA of cellular (**E**,**K**,**Q**) and secreted (**F**,**L**,**R**) IL1β protein (pg/mL) in hOC chondrocytes (**E**,**F**), hOB osteoblasts (**K**,**L**) and hSW synoviocytes (**Q**,**R**).

As such, we decided to further explore this potential mechanism of action in primary human OA joint cells. In TLR4/IL1R-activated hOC, gene expression (mRNA) of NLRP3, CASP1, and IL1β was highly increased (Figure 6A–C). In contrast, treatment with BBA, CLI-095, or siTLR4 reduced the induced mRNA levels (Figure 6A–C). Interestingly, NLRP3 silencing with siRNA (siNLRP3) recapitulated the blocking effects of BBA, CLI-095, and siTLR4 in TLR4/IL1R-activated hOC (Figure 6A–C). Furthermore, CASP1 activity assay and IL1β ELISA protein assay were used to validate these results (Figure 6D–F). Stimulation with LPS or IL1β increased CASP1 activity and cellular and secreted IL1β protein (Figure 6D–F). In line with previous results, treatment with BBA and CLI-095 reduced CASP1 activity and cellular and secreted IL1β levels to basal levels (Figure 6D–F).

The NLRP3 pathway plays a relevant role in bone differentiation and inflammation [102]. The TLR4/IL1R activation of hOB increased mRNA levels of NLRP3, CASP1, and IL1β (Figure 6G–I). Moreover, this activation also increased CASP1 activity and cellular and secreted IL1β protein levels (Figure 6J–L). As observed in hOC, treatment with TLR4 inhibitors BBA, CLI-095, or siTLR4, decreased NLRP3 expression, as well as CASP1 activity and IL1β protein levels (Figure 6G–L). Remarkably, siNLRP3 recapitulated the effects of TLR4 inhibition by reducing the mRNA levels of NLRP3, CASP1, and IL1β (Figure 6G–L). Likewise, the NLRP3 pathway is critical for synovial inflammation. Consistent with this, TLR4/IL1R-activated hSW synoviocytes exhibited increased mRNA levels of NLRP3, CASP1, and IL1β, increased CASP1 activity, and increased cellular and secreted IL1β levels (Figure 6M–R). Treatment with TLR4 inhibitors BBA, CLI-095, siTLR4, and/or siNLRP3 reduced all the studied NLRP3 pathway elements (Figure 6M–R). Interestingly, these effects were also observed in non-activated joint cells (chondrocytes, osteoblasts, and synoviocytes) (Figure 6A–C).

**Figure 7:**
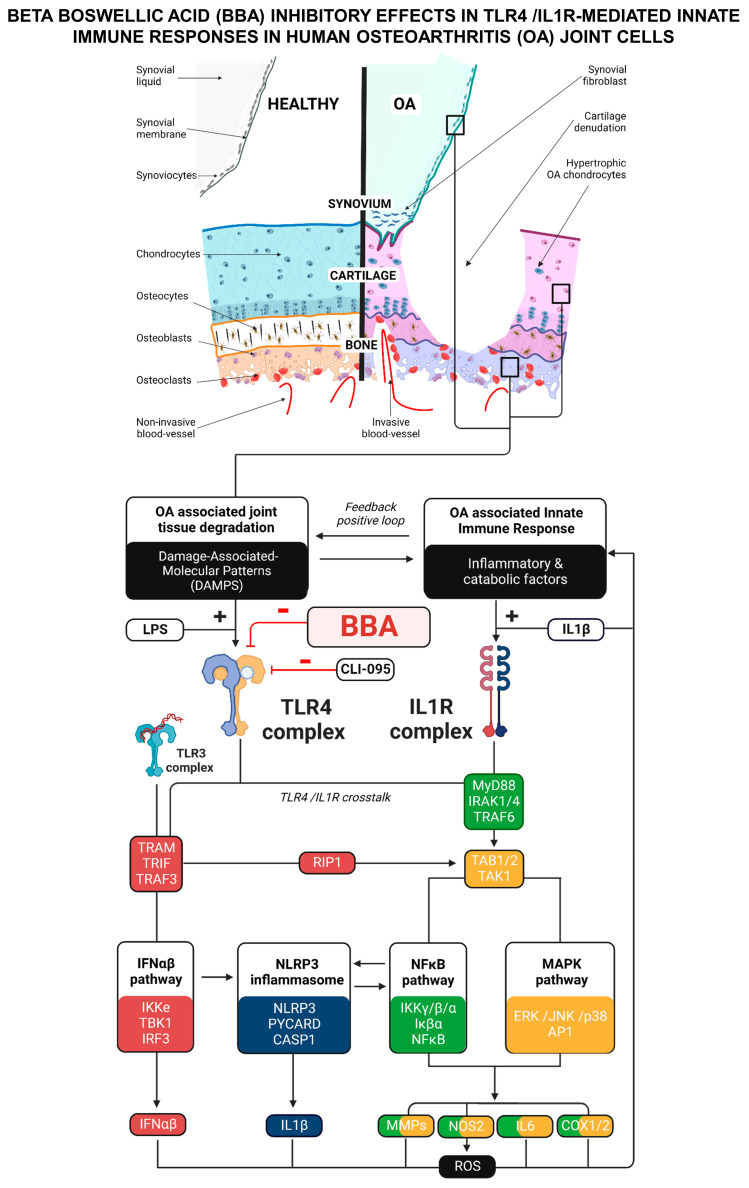
Diagram of TLR4/IL1R signaling, MAPK p38/NFκB, IFN, TNF, and NLRP3 downstream pathways and innate immune response (IIR) in osteoarthritis (OA) joint cells.

All in all, the mechanism of action of BBA inhibitory effects on TLR4/IL1R-mediated IIR has proven to be directly associated with the NLRP3 pathway as well as MAPK p38 and NFκB, IFN, and TNF pathways (Figure 7). Furthermore, BBA inhibitory effects might also come from the partial interruption of the positive feedback between IIR factors, ROS, DAMPs/PAMPs, and the TLR4/IL1R crosstalk (Figure 7).

## 4. Discussion

We present evidence that β boswellic acid or BBA selectively binds to the TLR4 complex and inhibits in primary human joint cells the TLR4/lL1R-mediated innate immune responses associated with the activation of ROS synthesis, MAPK p38/NFκB, IFNαβ, TNF, and NLRP3 signaling pathways. Furthermore, we determined in stimulated and non-stimulated articular cells that TLR4 inhibition by BBA or other known inhibitors is associated with NLRP3 downregulation. Moreover, we revealed that this downregulation was enough to recapitulate the effects of BBA or TLR4 inhibition on TLR4/lL1R-mediated IIR. Altogether, we provided relevant data supporting BBA as a strong TLR4 inhibitor that may help to control OA-associated IIR.

The BBA is extracted from *Boswellia serrata* and it is present in the BSE mix of triterpenes, essential oils, and polysaccharides that it is commonly taken as an infusion or food complement in traditional ayurvedic medicine to treat inflammatory diseases such as asthma, inflammatory bowel disease, RA, and OA. Interestingly, the evidence failed to demonstrate that BSE anti-inflammatory effects were a consequence of 5-lipoxygenase [27] inhibition. On the contrary, the BSE element BBA has recently been described to inhibit NFκB, a downstream signaling pathway of TLR4/IL1R [103,104]. Remarkably, TLR4 and IL1R are two innate immune receptors associated with the development of multiple rheumatic diseases [17,38,39,40,41,50,51,52]. The IL1R has been used as a therapeutic target for diseases such as OA [17], RA [50], or gout [105]. However, there is no clinically available TLR4 inhibitor to treat rheumatic inflammatory diseases [22,106]. Considering the traditional use of BSE as antirheumatic and the new potential properties of BBA, we decided to determine whether BBA was able to block TLR4 and inhibit OA-associated IIR in joint cells.

Considering that a functional TLR4 receptor is required for full IL1R signaling, the first approach was to analyze the primary human OA chondrocytes secretome and proteome (Figure 1) changes linked to TLR4 and IL1R activation [22]. We obtained key data on the TLR4/IL1R downstream signaling pathways. Specifically, MAPK p38/NFκB, TNF, IFNαβ, ROS, and NLRP3 downstream pathways were found enriched whereas ECM anabolic pathways were found depleted in the proteome of stimulated chondrocytes. These proteomic changes were consistent with increased inflammatory and catabolic OA-like phenotype [107,108,109]. Likewise, the enrichment in ROS-related pathways including NO synthesis was in concordance with OA worsening associated with oxidative stress [110,111]. Similar results were obtained in mice ATDC5 chondrocytes proteome (Figure S1).

Next, through computational pharmacology, we investigated BBA potential inhibition of key factors implicated in major pathways found in the secretome and proteome of TLR4/IL1R-activated primary human OA chondrocytes (Figure 2). We predicted a greater binding affinity of BBA to the human TLR4 complex than other elements suggested in the literature such as NFκB [93] and IKK [66,112]. Furthermore, this binding affinity of BBA to TLR4 was found equivalent to known antagonists such as CLI-095. Interestingly, the docking analysis predicted that BBA could not bind to any domain of IL1R, NLRP3, or TLR3 complex. Although docking score results found the binding of BBA to TLR4 the most energetically and structurally stable, the BBA effective permeability coefficient does not discard BBA from crossing the cellular membrane and binding to NFκB, as the literature suggested. Nonetheless, molecular dynamics simulations presented additional evidence of BBA-TLR4 binding stability, suggesting that specific TLR4 inhibition may be the main mechanism of action (Figure 2).

The combination of anti-TLR4 predicted activity of BBA and the TLR4/IL1R targets identified from the proteome analysis supported the in vitro study of BBA effects in TLR4/IL1R-activated human chondrocytes, osteoblasts, and synoviocytes. The relevance of this predicted BBA activity was underpinned by the observation of increased levels of OA-associated factors as well as increased sensitivity to TLR4 and IL1R agonists in chondrocytes, osteoblasts, and synoviocytes isolated from OA patients in contraposition to healthy ones.

Data obtained in all these cells revealed that BBA cotreatment reduced the gene expression of factors related to MAPK p38/NFκB (IL6, LCN2, COX1, COX2, ICAM1, VCAM1, MCP1, and NAMPT), ECM catabolism (MMP-1, -3, -9, -13, and ADAMTS4), TNF, IFN, NLRP3 inflammasome (NLRP3, CASP1, IL1β), and ROS pathways in a dose-response manner. In contrast, BBA cotreatment restored the ECM anabolism loss after TLR/IL1R activation. Supporting these results, the protein expression of key cytokines and the phosphoproteome of these stimulated cells was significantly reduced by BBA anti-TLR4 effect. Furthermore, BBA activity did not cause negative effects on cell vitality. Similar results were obtained in mice ATDC5 chondrocytes.

Considering that the specific TLR4 inhibitor CLI-095 and the anti-TLR4 siRNA replicated the same BBA inhibitory effects in all the cellular studies [113], and neither CLI-095 nor BBA downregulated TLR4 and IL1R expression, it was confirmed that TLR4 inhibition is the mechanism underlying BBA effect. In agreement with this, previous reports with other TLR4 inhibitors have obtained similar results about the key role of TLR4 inhibition on IL1R signaling [22,23]. Remarkably, BBA downregulated basal expression levels of several IIR factors suggesting that TLR4 constitutive activation contributes to the expression of multiple IIR factors in joint cells.

Despite BBA not binding to to IL1R, it exhibited outstanding effects in blocking IL1R-mediated IIR. This was consistent with the described anti-inflammatory effects of TLR4 inhibition on the IL1-mediated RA animal model [114]. The NLRP3 inflammasome pathway has been associated with the IIR in multiple rheumatic diseases [115,116], including OA [117]. Moreover, this pathway was substantially enriched in chondrocytes upon the activation of TLR4 and ILR1 receptors. In line with this, the NLRP3 inflammasome pathway has been identified downstream of TLR4/IL1R crosstalk [117,118]. Specifically, it participates in the synthesis and release of IL1β, which generates DAMPs that activate TLR4 [17] promoting an inflammatory positive feedback loop. Accordingly, we investigated whether NLRP3 signaling pathway modulation could be responsible for BBA effects. Interestingly, in all the articular cells studied, BBA was able to downregulate the TLR/IL1R-induced expression of the NLRP3 axis (NLRP3, CASP1, and IL1β) as well as the enzymatic activity of CASP1, and the concentration of cellular and secreted IL1β protein. Similar results were obtained in non-activated OA joint cells. Consistent with this, NLRP3 and TLR4 silencing recapitulated the inhibitory effects of BBA and CLI-095 in OA joint cells. These data demonstrated that NLRPL3 signaling is tightly related to TLR4 and confirmed that BBA effects on IL1R signaling are TLR4- and NLRP3-dependent. Overall, the local use of BBA on the articular tissues might be open to consideration.

## 5. Conclusions

We demonstrate that BBA inhibits OA-associated inflammatory and catabolic processes in chondrocytes, osteoblasts, and synoviocytes without altering the joint cell viability. We prove that the specific binding of BBA to the TLR4 complex inhibits TLR4 and IL1R signaling and downregulates MAPK p38/NFκB, NLRP3 inflammasome, and other OA-associated pathways. Moreover, we identified the TLR4-NLRP3 axis as an underlying mechanism of the BBA effect on IL1R signaling. Altogether, we present a solid bulk of evidence that BBA blocks OA innate immune responses and could be transferred into the clinic as an alimentary supplement or as a therapeutic tool after clinical trial evaluations.

## Data Availability

The data presented in this study are available in the article and Supplementary Materials.

## Supplementary Materials

The following are available online at https://www.mdpi.com/article/10.3390/antiox12020371/s1, Figure S1: BBA effects on the proteome and transcriptome of TLR4/IL1R-mediated innate immune responses in mouse ATDC5 chondrocytes: (**A**–**E**) Proteome profile by DDA MALDI-TOFF of mice ATDC5 chondrocytes stimulated with LPS (100 ng/mL) or IL1β (0.1 ng/mL) for 48 h. Independent biological replicas (*n* = 3) were normalized (control) and expressed as the mean ± SEM; (**A**) Mice proteome profile of non-filtered (FDR ≤ 0% = 5772) and filtered (FDR ≤ 5% = 3328) cellular proteins; (**B**) Venn diagram (FDR ≤ 5%) of TLR4 (LPS)/IL1R (IL1β)-activated and non-activated (Control) mice ATDC5 chondrocytes proteome; (**C**) Interactome plot (FDR ≤ 5%) of TLR4/IL1R-activated mice ATDC5 chondrocytes proteome; (**D**,**E**) Pathway Enrichment Analysis (FDR ≤ 5%; fold-change) of TLR4/IL1R-activated mice ATDC5 chondrocytes proteome. (**F**–**N**) Mouse ATDC5 chondrocytes were treated with BBA (0.10–5.00 µM), CLI-095 (1 µM) or siTLR4 (15 nM) for 24 h and cotreated with LPS (100 ng/mL) or IL1β (0.1 ng/mL) for another 48 h. Independent biological replicas (*n* = 6) were normalized (control) and expressed as the mean ± SEM; (**F**,**G**) MTT cell viability assay in ATDC5 chondrocytes displaying cell inviable doses (red), cell-viable doses (green) and FBS as a positive control; (**H**,**I**) Griess—ROS assay measuring NO_2^−^_ levels in ATDC5 chondrocytes; (**J**–**N**): RT-qPCR gene expression (mRNA) of NOS2, TLR4, IL1R, TNFα and IFNB1 in ATDC5 chondrocytes; Figure S2: BBA effects on the transcriptome of TLR4/IL1R-mediated innate immune responses in mouse ATDC5 chondrocytes: (**A**–**U**) Mouse ATDC5 chondrocytes were treated with BBA (0.10–5.00 µM), CLI-095 (1 µM) or siTLR4 (15 nM) for 24 h and cotreated with LPS (100 ng/mL) or IL1β (0.1 ng/mL) for another 48 h. Independent biological replicas (*n* = 6) were normalized (control) and expressed as the mean ± SEM. (**A**–**R**) RT-qPCR gene expression (mRNA) of IL6, LCN2, COX1, COX2, ICAM1, VCAM1, MCP1, NAMPT, MMP-1, -3, -9, -13, ADAMTS4, ACAN, COL2α1, NLRP3, CASP1 and IL1β in ATDC5 chondrocytes; (**S**) CASP1 activity (pNa reaction) assay in mice ATDC5 chondrocytes; (**T**–**U**): ELISA of cellular (**T**) and secreted (**U**) IL1β protein (pg/mL) in mice ATDC5 chondrocytes.

## References

[1] World Health Organization Disease Incidence, Prevalence and Disability (2004). The Global Burden of Disease: 2004 Update.

[2] United Nations World Population Prospects—Population Division—United Nations. https://population.un.org/wpp/.

[3] Thomas E., Peat G., Croft P. (2014). Defining and Mapping the Person with Osteoarthritis for Population Studies and Public Health. Rheumatology.

[4] Oo W.M., Yu S.P.-C., Daniel M.S., Hunter D.J. (2018). Disease-Modifying Drugs in Osteoarthritis: Current Understanding and Future Therapeutics. Expert Opin. Emerg. Drugs.

[5] Mobasheri A., Saarakkala S., Finnilä M., Karsdal M.A., Bay-Jensen A.-C., van Spil W.E. (2019). Recent Advances in Understanding the Phenotypes of Osteoarthritis. F1000Research.

[6] Karsdal M.A., Michaelis M., Ladel C., Siebuhr A.S., Bihlet A.R., Andersen J.R., Guehring H., Christiansen C., Bay-Jensen A.C., Kraus V.B. (2016). Disease-Modifying Treatments for Osteoarthritis (DMOADs) of the Knee and Hip: Lessons Learned from Failures and Opportunities for the Future. Osteoarthr. Cartil..

[7] Hunter D.J., Bierma-Zeinstra S. (2019). Osteoarthritis. Lancet.

[8] Vina E.R., Kwoh C.K. (2018). Epidemiology of Osteoarthritis: Literature Update. Curr. Opin. Rheumatol..

[9] Schaible H.-G. (2018). Osteoarthritis Pain. Recent Advances and Controversies. Curr. Opin. Support. Palliat. Care.

[10] Li K., Zhang Y., Zhang Y., Jiang W., Shen J., Xu S., Cai D., Shen J., Huang B., Li M. (2018). Tyrosine Kinase Fyn Promotes Osteoarthritis by Activating the β-Catenin Pathway. Ann. Rheum. Dis..

[11] Zhou Y., Wang T., Hamilton J.L., Chen D. (2017). Wnt/β-Catenin Signaling in Osteoarthritis and in Other Forms of Arthritis. Curr. Rheumatol. Rep..

[12] Saito T., Tanaka S. (2017). Molecular Mechanisms Underlying Osteoarthritis Development: Notch and NF-ΚB. Arthritis. Res. Ther..

[13] Berenbaum F. (2013). Osteoarthritis as an Inflammatory Disease (Osteoarthritis Is Not Osteoarthrosis!). Osteoarthr. Cartil..

[14] Mathiessen A., Conaghan P.G. (2017). Synovitis in Osteoarthritis: Current Understanding with Therapeutic Implications. Arthritis. Res. Ther..

[15] Roelofs A.J., Kania K., Rafipay A.J., Sambale M., Kuwahara S.T., Collins F.L., Smeeton J., Serowoky M.A., Rowley L., Wang H. (2020). Identification of the Skeletal Progenitor Cells Forming Osteophytes in Osteoarthritis. Ann. Rheum. Dis..

[16] Kovács B., Vajda E., Nagy E.E. (2019). Regulatory Effects and Interactions of the Wnt and OPG-RANKL-RANK Signaling at the Bone-Cartilage Interface in Osteoarthritis. Int. J. Mol. Sci..

[17] Gómez R., Villalvilla A., Largo R., Gualillo O., Herrero-Beaumont G. (2014). TLR4 Signalling in Osteoarthritis-Finding Targets for Candidate DMOADs. Nat. Rev. Rheumatol..

[18] Sohn D., Sokolove J., Sharpe O., Erhart J.C., Chandra P.E., Lahey L.J., Lindstrom T.M., Hwang I., Boyer K.A., Andriacchi T.P. (2012). Plasma Proteins Present in Osteoarthritic Synovial Fluid Can Stimulate Cytokine Production via Toll-like Receptor 4. Arthritis. Res. Ther..

[19] Rousseau J.C., Garnero P. (2012). Biological Markers in Osteoarthritis. Bone.

[20] Litwic A., Registrar S., Edwards M., Clinical M. (2013). Europe PMC Funders Group Epidemiology and Burden of Osteoarthritis. Epidemiology.

[21] Sinyeue C., Matsui M., Oelgemöller M., Bregier F., Chaleix V., Sol V., Lebouvier N. (2022). Synthesis and Investigation of Flavanone Derivatives as Potential New Anti-Inflammatory Agents. Molecules.

[22] Franco-Trepat E., Alonso-Pérez A., Guillán-Fresco M., Jorge-Mora A., Crespo-Golmar A., López-Fagúndez M., Pazos-Pérez A., Gualillo O., Belén Bravo S., Gómez Bahamonde R. (2022). Amitriptyline Blocks Innate Immune Responses Mediated by Toll-like Receptor 4 and IL-1 Receptor: Preclinical and Clinical Evidence in Osteoarthritis and Gout. Br. J. Pharmacol..

[23] Franco-Trepat E., Guillán-Fresco M., Alonso-Pérez A., López-Fagúndez M., Pazos-Pérez A., Crespo-Golmar A., Gualillo O., Jorge-Mora A., Bravo S.B., Gómez R. (2022). Repurposing Drugs to Inhibit Innate Immune Responses Associated with TLR4, IL1, and NLRP3 Signaling in Joint Cells. Biomed. Pharm..

[24] Yang M., Jiang L., Wang Q., Chen H., Xu G. (2017). Traditional Chinese Medicine for Knee Osteoarthritis: An Overview of Systematic Review. PLoS ONE.

[25] Kessler C.S., Pinders L., Michalsen A., Cramer H. (2015). Ayurvedic Interventions for Osteoarthritis: A Systematic Review and Meta-Analysis. Rheumatol. Int..

[26] Pathania M., Bhardwaj P., Pathania N., Rathaur V. (2020). A Review on Exploring Evidence-Based Approach to Harnessing the Immune System in Times of Corona Virus Pandemic: Best of Modern and Traditional Indian System of Medicine. J. Fam. Med. Prim. Care.

[27] Wang Q., Pan X., Wong H.H., Wagner C.A., Lahey L.J., Robinson W.H., Sokolove J. (2014). Oral and Topical Boswellic Acid Attenuates Mouse Osteoarthritis. Osteoarthr. Cartil..

[28] Efferth T., Oesch F. (2022). Anti-Inflammatory and Anti-Cancer Activities of Frankincense: Targets, Treatments and Toxicities. Semin. Cancer Biol..

[29] Shah S.A., Rathod I.S., Suhagia B.N., Patel D.A., Parmar V.K., Shah B.K., Vaishnavi V.M. (2007). Estimation of Boswellic Acids from Market Formulations of Boswellia Serrata Extract and 11-Keto Beta-Boswellic Acid in Human Plasma by High-Performance Thin-Layer Chromatography. J. Chromatogr. B Analyt. Technol. Biomed. Life Sci..

[30] Gerbeth K., Meins J., Kirste S., Momm F., Schubert-Zsilavecz M., Abdel-Tawab M. (2011). Determination of Major Boswellic Acids in Plasma by High-Pressure Liquid Chromatography/Mass Spectrometry. J. Pharm. Biomed. Anal..

[31] Riva A., Allegrini P., Franceschi F., Togni S., Giacomelli L., Eggenhoffner R. (2017). A Novel Boswellic Acids Delivery Form (Casperome®) in the Management of Musculoskeletal Disorders: A Review. Eur. Rev. Med. Pharmacol. Sci..

[32] Gupta S., Ahsan A.U., Wani A., Khajuria V., Nazir L.A., Sharma S., Bhagat A., Raj Sharma P., Bhardwaj S., Peerzada K.J. (2018). The Amino Analogue of β-Boswellic Acid Efficiently Attenuates the Release of pro-Inflammatory Mediators than Its Parent Compound through the Suppression of NF-ΚB/IκBα Signalling Axis. Cytokine.

[33] Abdel-Tawab M., Werz O., Schubert-Zsilavecz M. (2011). Boswellia Serrata: An Overall Assessment of in Vitro, Preclinical, Pharmacokinetic and Clinical Data. Clin. Pharmacokinet..

[34] Thummuri D., Jeengar M.K., Shrivastava S., Areti A., Yerra V.G., Yamjala S., Komirishetty P., Naidu V.G.M., Kumar A., Sistla R. (2014). Boswellia Ovalifoliolata Abrogates ROS Mediated NF-ΚB Activation, Causes Apoptosis and Chemosensitization in Triple Negative Breast Cancer Cells. Environ. Toxicol. Pharmacol..

[35] Umar S., Umar K., Sarwar A.H.M.G., Khan A., Ahmad N., Ahmad S., Katiyar C.K., Husain S.A., Khan H.A. (2014). Boswellia Serrata Extract Attenuates Inflammatory Mediators and Oxidative Stress in Collagen Induced Arthritis. Phytomedicine.

[36] Kimmatkar N., Thawani V., Hingorani L., Khiyani R. (2003). Efficacy and Tolerability of Boswellia Serrata Extract in Treatment of Osteoarthritis of Knee--a Randomized Double Blind Placebo Controlled Trial. Phytomedicine.

[37] Majeed M., Majeed S., Narayanan N.K., Nagabhushanam K., Narayanan |., Narayanan K., Nagabhushanam K. (2019). A Pilot, Randomized, Double-Blind, Placebo-Controlled Trial to Assess the Safety and Efficacy of a Novel Boswellia Serrata Extract in the Management of Osteoarthritis of the Knee. Phytother. Res..

[38] Samarpita S., Kim J.Y., Rasool M.K., Kim K.S. (2020). Investigation of Toll-like Receptor (TLR) 4 Inhibitor TAK-242 as a New Potential Anti-Rheumatoid Arthritis Drug. Thromb. Haemost..

[39] Loiarro M., Ruggiero V., Sette C. (2010). Targeting TLR/IL-1R Signalling in Human Diseases. Mediat. Inflamm..

[40] Mitchell S., Vargas J., Hoffmann A. (2016). Signaling via the NFκB System. WIREs Syst. Biol. Med..

[41] Frazão J.B., Errante P.R., Condino-Neto A. (2013). Toll-Like Receptors’ Pathway Disturbances Are Associated with Increased Susceptibility to Infections in Humans. Arch. Immunol. Ther. Exp..

[42] Abbaszade I., Liu R.Q., Yang F., Rosenfeld S.A., Ross O.H., Link J.R., Ellis D.M., Tortorella M.D., Pratta M.A., Hollist J.M. (1999). Cloning and Characterization of ADAMTS11, an Aggrecanase from the ADAMTS Family. J. Biol. Chem..

[43] Van Den Berg W.B., Van De Loo F.A.J., Zwarts W.A., Otterness I.G. (1988). Effects of Murine Recombinant Interleukin 1 on Intact Homologous Articular Cartilage: A Quantitative and Autoradiographic Study. Ann. Rheum. Dis..

[44] Van de Loo A.A.J., Van den Berg W.B. (1990). Effects of Murine Recombinant Interleukin 1 on Synovial Joints in Mice: Measurement of Patellar Cartilage Metabolism and Joint Inflammation. Ann. Rheum. Dis..

[45] Lefebvre V., Peeters-Joris C., Vaes G. (1990). Modulation by Interleukin 1 and Tumor Necrosis Factor α of Production of Collagenase, Tissue Inhibitor of Metalloproteinases and Collagen Types in Differentiated and Dedifferentiated Articular Chondrocytes. BBA Mol. Cell Res..

[46] Balavoine J.F., de Rochemonteix B., Williamson K. (1986). Prostaglandin E2 and Collagenase Production by Fibroblasts and Synovial Cells Is Regulated by Urine-Derived Human Interleukin 1 and Inhibitor(s). J. Clin. Investig..

[47] Gadher S.J., Eyre D.R., Duance V.C., Wotton S.F., Heck L.W., Schmid T.M., Woolley D.E. (1988). Susceptibility of Cartilage Collagens Type II, IX, X, and XI to Human Synovial Collagenase and Neutrophil Elastase. Eur. J. Biochem..

[48] Tyler J.A. (1985). Articular Cartilage Cultured with Catabolin (Pig Interleukin 1) Synthesizes a Decreased Number of Normal Proteoglycan Molecules. Biochem. J..

[49] Tortorella M.D., Burn T.C., Pratta M.A., Abbaszade I., Hollis J.M., Liu R., Rosenfeld S.A., Copeland R.A., Decicco C.P., Wynn R. (1999). Purification and Cloning of Aggrecanase-1: A Member of the ADAMTS Family of Proteins. Science.

[50] Mertens M., Singh J.A. (2009). Anakinra for Rheumatoid Arthritis. Cochrane Database Syst. Rev..

[51] Vincent T.L. (2019). IL-1 in Osteoarthritis: Time for a Critical Review of the Literature. F1000Research.

[52] Alonso-Pérez A., Franco-Trepat E., Guillán-Fresco M., Jorge-Mora A., López V., Pino J., Gualillo O., Gómez R. (2018). Role of Toll-Like Receptor 4 on Osteoblast Metabolism and Function. Front. Physiol..

[53] Rigoglou S., Papavassiliou A.G. (2013). The NF-ΚB Signalling Pathway in Osteoarthritis. Int. J. Biochem. Cell Biol..

[54] Conde J., Gomez R., Bianco G., Scotece M., Lear P., Dieguez C., Gomez-Reino J., Lago F., Gualillo O. (2011). Expanding the Adipokine Network in Cartilage: Identification and Regulation of Novel Factors in Human and Murine Chondrocytes. Ann. Rheum. Dis..

[55] Gómez R., Conde J., Scotece M., Gómez-Reino J.J., Lago F., Gualillo O. (2011). What’s New in Our Understanding of the Role of Adipokines in Rheumatic Diseases?. Nat. Rev. Rheumatol..

[56] Choi M.-C., Jo J., Park J., Kang H.K., Park Y. (2019). NF-B Signaling Pathways in Osteoarthritic Cartilage Destruction. Cells.

[57] Bai F., Chen X., Yang H., Xu H.G. (2018). Acetyl-11-Keto-β-Boswellic Acid Promotes Osteoblast Differentiation by Inhibiting Tumor Necrosis Factor-α and Nuclear Factor-ΚB Activity. J. Craniofacial Surg..

[58] Al-Bahlani S., Burney I.A., Al-Dhahli B., Al-Kharusi S., Al-Kharousi F., Al-Kalbani A., Ahmed I. (2020). Boswellic Acid Sensitizes Gastric Cancer Cells to Cisplatin-Induced Apoptosis via P53-Mediated Pathway. BMC Pharmacol. Toxicol..

[59] Ranzato E., Martinotti S., Volante A., Tava A., Masini M.A., Burlando B. (2017). The Major Boswellia Serrata Active 3-Acetyl-11-Keto-β-Boswellic Acid Strengthens Interleukin-1α Upregulation of Matrix Metalloproteinase-9 via JNK MAP Kinase Activation. Phytomedicine.

[60] Goswami D., Das Mahapatra A., Banerjee S., Kar A., Ojha D., Mukherjee P.K., Chattopadhyay D. (2018). Boswellia Serrata Oleo-Gum-Resin and β-Boswellic Acid Inhibits HSV-1 Infection in Vitro through Modulation of NF-KB and P38 MAP Kinase Signaling. Phytomedicine.

[61] Han C., Yu X., Zhang C., Cai Y., Cao Y., Wang S., Shen J. (2019). Drug Repurposing Screen Identifies Novel Classes of Drugs with Anticancer Activity in Mantle Cell Lymphoma. Comb. Chem. High Throughput Screen..

[62] Park K. (2019). A Review of Computational Drug Repurposing. Transl. Clin. Pharmacol..

[63] Yang G., Lee H.S.E., Moon S., Ko K.M., Koh J.H., Seok J.K., Min J., Heo T., Kang H.C., Cho Y. (2020). Direct Binding to NLRP3 Pyrin Domain as a Novel Strategy to Prevent NLRP3-Driven Inflammation and Gouty Arthritis. Arthritis Rheumatol..

[64] PubChem. https://pubchem.ncbi.nlm.nih.gov/.

[65] Burley S.K., Berman H.M., Bhikadiya C., Bi C., Chen L., Di Costanzo L., Christie C., Dalenberg K., Duarte J.M., Dutta S. (2019). RCSB Protein Data Bank: Biological Macromolecular Structures Enabling Research and Education in Fundamental Biology, Biomedicine, Biotechnology and Energy. Nucleic Acids Res..

[66] Xu G., Lo Y.C., Li Q., Napolitano G., Wu X., Jiang X., Dreano M., Karin M., Wu H. (2011). Crystal Structure of Inhibitor of Κb Kinase β. Nature.

[67] Sharif H., Wang L., Wang W.L., Magupalli V.G., Andreeva L., Qiao Q., Hauenstein A.V., Wu Z., Núñez G., Mao Y. (2019). Structural Mechanism for NEK7-Licensed Activation of NLRP3 Inflammasome. Nature.

[68] Sandall C.F., Ziehr B.K., MacDonald J.A. (2020). ATP-Binding and Hydrolysis in Inflammasome Activation. Molecules.

[69] El-Sharkawy L.Y., Brough D., Freeman S. (2020). Inhibiting the Nlrp3 Inflammasome. Molecules.

[70] Sastry G.M., Adzhigirey M., Day T., Annabhimoju R., Sherman W. (2013). Protein and Ligand Preparation: Parameters, Protocols, and Influence on Virtual Screening Enrichments. J. Comput. Aided Mol. Des..

[71] Friesner R.A., Murphy R.B., Repasky M.P., Frye L.L., Greenwood J.R., Halgren T.A., Sanschagrin P.C., Mainz D.T. (2006). Extra Precision Glide: Docking and Scoring Incorporating a Model of Hydrophobic Enclosure for Protein-Ligand Complexes. J. Med. Chem..

[72] Halgren T.A., Murphy R.B., Friesner R.A., Beard H.S., Frye L.L., Pollard W.T., Banks J.L. (2004). Glide: A New Approach for Rapid, Accurate Docking and Scoring. 2. Enrichment Factors in Database Screening. J. Med. Chem..

[73] Jacobson M.P., Friesner R.A., Xiang Z., Honig B. (2002). On the Role of the Crystal Environment in Determining Protein Side-Chain Conformations. J. Mol. Biol..

[74] Jacobson M.P., Pincus D.L., Rapp C.S., Day T.J.F., Honig B., Shaw D.E., Friesner R.A. (2004). A Hierarchical Approach to All-Atom Protein Loop Prediction. Proteins Struct. Funct. Bioinform..

[75] Cerón-Carrasco J.P. (2022). When Virtual Screening Yields Inactive Drugs: Dealing with False Theoretical Friends. Chem. Med. Chem..

[76] Cerón-Carrasco J.P., Jacquemin D. (2021). Using Theory to Extend the Scope of Azobenzene Drugs in Chemotherapy: Novel Combinations for a Specific Delivery. ChemMedChem.

[77] Villalvilla A., Silva A., Largo R., Gualillo O. (2014). 6-Shogaol Inhibits Chondrocytes ’ Innate Immune Responses and Cathepsin-K Activity. Mol. Nutr. Food Res..

[78] Alonso-Pérez A., Guillán-Fresco M., Franco-Trepat E., Jorge-Mora A., López-Fagúndez M., Pazos-Pérez A., Crespo-Golmar A., Caeiro-Rey J.R., Gómez R. (2022). Improved Protocol to Study Osteoblast and Adipocyte Differentiation Balance. Biomedicines..

[79] Hussey S.E., Liang H., Costford S.R., Klip A., DeFronzo R.A., Sanchez-Avila A., Ely B., Musi N. (2012). TAK-242, a Small-Molecule Inhibitor of Toll-like Receptor 4 Signalling, Unveils Similarities and Differences in Lipopolysaccharide- and Lipid-Induced Inflammation and Insulin Resistance in Muscle Cells. Biosci. Rep..

[80] Hannon G.J., Rossi J.J. (2004). Unlocking the Potential of the Human Genome with RNA Interference. Nature.

[81] Omega Biotek E.Z.N. (2012). A Total RNA Kit I Product Manual. https://www.omegabiotek.com/wp-content/uploads/2017/08/R6834-Quick-Guide.pdf.

[82] (2018). AppliedBiosystems High-Capacity RNA-to-CDNA Kit Product Information Sheet. https://tools.thermofisher.com/content/sfs/manuals/4387949_RNAtocDNA_PI.pdf.

[83] Bustin S.A., Benes V., Garson J.A., Hellemans J., Huggett J., Kubista M., Mueller R., Nolan T., Pfaffl M.W., Shipley G.L. (2009). The MIQE Guidelines: Minimum Information for Publication of Quantitative Real-Time PCR Experiments. Clin. Chem..

[84] QuantStudio 3 and 5 Real-Time PCR System Software|Thermo Fisher Scientific—ES. https://www.thermofisher.com/es/es/home/global/forms/life-science/quantstudio-3-5-software.html.

[85] QuantStudio Design & Analysis. https://apps.thermofisher.com/apps/da2/#/home/welcome.

[86] Couselo-Seijas M., López-Canoa J.N., Agra-Bermejo R.M., Díaz-Rodriguez E., Fernandez A.L., Martinez-Cereijo J.M., Durán-Muñoz D., Bravo S.B., Velo A., González-Melchor L. (2019). Cholinergic Activity Regulates the Secretome of Epicardial Adipose Tissue: Association with Atrial Fibrillation. J. Cell. Physiol..

[87] López-Pedrouso M., Franco D., Serrano M.P., Maggiolino A., Landete-Castillejos T., De Palo P., Lorenzo J.M. (2019). A Proteomic-Based Approach for the Search of Biomarkers in Iberian Wild Deer (Cervus Elaphus) as Indicators of Meat Quality. J. Proteom..

[88] UniProt Consortium (2019). UniProt: A Worldwide Hub of Protein Knowledge. Nucleic Acids Res..

[89] Shilov I.V., Seymour S.L., Patel A.A., Loboda A., Tang W.H., Keating S.P., Hunter C.L., Nuwaysir L.M., Schaeffer D.A. (2007). The Paragon Algorithm, a Next Generation Search Engine That Uses Sequence Temperature Values and Feature Probabilities to Identify Peptides from Tandem Mass Spectra. Mol. Cell. Proteom..

[90] Fabregat A., Jupe S., Matthews L., Sidiropoulos K., Gillespie M., Garapati P., Haw R., Jassal B., Korninger F., May B. (2018). The Reactome Pathway Knowledgebase. Nucleic Acids Res..

[91] Pathan M., Keerthikumar S., Chisanga D., Alessandro R., Ang C.S., Askenase P., Batagov A.O., Benito-Martin A., Camussi G., Clayton A. (2017). A Novel Community Driven Software for Functional Enrichment Analysis of Extracellular Vesicles Data. J. Extracell. Vesicles.

[92] Komander D. (2009). The Emerging Complexity of Protein Ubiquitination. Biochem. Soc. Trans..

[93] Müller C.W., Harrison S.C. (1995). The Structure of the NF-Kappa B P50:DNA-Complex: A Starting Point for Analyzing the Rel Family. FEBS Lett..

[94] Fitzgerald K.A., Kagan J.C. (2020). Toll-like Receptors and the Control of Immunity. Cell.

[95] Ohto U., Fukase K., Miyake K., Shimizu T. (2012). Structural Basis of Species-Specific Endotoxin Sensing by Innate Immune Receptor TLR4/MD-2. Proc. Natl. Acad. Sci. USA.

[96] Tapia-Abellán A., Angosto-Bazarra D., Martínez-Banaclocha H., de Torre-Minguela C., Cerón-Carrasco J.P., Pérez-Sánchez H., Arostegui J.I., Pelegrin P. (2019). MCC950 Closes the Active Conformation of NLRP3 to an Inactive. Nat. Chem. Biol..

[97] Chi C.T., Lee M.H., Weng C.F., Leong M.K. (2019). In Silico Prediction of PAMPA Effective Permeability Using a Two-QSAR Approach. Int. J. Mol. Sci..

[98] Dixon S.L., Duan J., Smith E., von Bargen C.D., Sherman W., Repasky M.P. (2016). AutoQSAR: An Automated Machine Learning Tool for Best-Practice Quantitative Structure-Activity Relationship Modeling. Futur. Med. Chem..

[99] Cernanec J.M., Weinberg J.B., Batinic-Haberle I., Guilak F., Fermor B. (2007). Influence of Oxygen Tension on Interleukin 1-Induced Peroxynitrite Formation and Matrix Turnover in Articular Cartilage. J. Rheumatol..

[100] Davies C.M., Guilak F., Weinberg J.B., Fermor B. (2008). Reactive Nitrogen and Oxygen Species in Interleukin-1-Mediated DNA Damage Associated with Osteoarthritis. Osteoarthr. Cartil..

[101] Whiteman M., Spencer J.P.E., Zhu Y.Z., Armstrong J.S., Schantz J.T. (2006). Peroxynitrite-Modified Collagen-II Induces P38/ERK and NF-ΚB-Dependent Synthesis of Prostaglandin E2 and Nitric Oxide in Chondrogenically Differentiated Mesenchymal Progenitor Cells. Osteoarthr. Cartil..

[102] Zhou F., Zhang G., Wu Y., Xiong Y. (2022). Inflammasome Complexes: Crucial Mediators in Osteoimmunology and Bone Diseases. Int. Immunopharmacol..

[103] Li X., Qin J. (2005). Modulation of Toll?Interleukin 1 Receptor Mediated Signaling. J. Mol. Med..

[104] Martin M.U., Wesche H. (2002). Summary and Comparison of the Signaling Mechanisms of the Toll/Interleukin-1 Receptor Family. Biochim. Biophys. Acta BBA Mol. Cell Res..

[105] Pascart T., Richette P. (2017). Current and Future Therapies for Gout. Expert Opin. Pharmacother..

[106] Park H., Hong J., Yin Y., Joo Y., Kim Y., Shin J., Kwon H.H., Shin N., Shin H.J., Beom J. (2020). TAP2, a Peptide Antagonist of Toll-like Receptor 4, Attenuates Pain and Cartilage Degradation in a Monoiodoacetate-Induced Arthritis Rat Model. Sci. Rep..

[107] Ha S.H., Kim H.K., Anh N.T.T., Kim N., Ko K.S., Rhee B.D., Han J. (2017). Time-Dependent Proteomic and Genomic Alterations in Toll-like Receptor-4-Activated Human Chondrocytes: Increased Expression of Lamin A/C and Annexins. Korean J. Physiol. Pharmacol..

[108] Haglund L., Bernier S.M., Önnerfjord P., Recklies A.D. (2008). Proteomic Analysis of the LPS-Induced Stress Response in Rat Chondrocytes Reveals Induction of Innate Immune Response Components in Articular Cartilage. Matrix Biol..

[109] Tardif G., Paré F., Gotti C., Roux-Dalvai F., Droit A., Zhai G., Sun G., Fahmi H., Pelletier J.-P., Martel-Pelletier J. (2022). Mass Spectrometry-Based Proteomics Identify Novel Serum Osteoarthritis Biomarkers. Arthritis. Res. Ther..

[110] Lepetsos P., Papavassiliou A.G. (2016). ROS/Oxidative Stress Signaling in Osteoarthritis. Biochim. Biophys. Acta.

[111] Coryell P.R., Diekman B.O., Loeser R.F. (2021). Mechanisms and Therapeutic Implications of Cellular Senescence in Osteoarthritis. Nat. Rev. Rheumatol..

[112] Gilmore T.D. (2006). Introduction to NF-KappaB: Players, Pathways, Perspectives. Oncogene.

[113] Krasselt M., Baerwald C. (2019). Celecoxib for the Treatment of Musculoskeletal Arthritis. Expert Opin. Pharmacother..

[114] Abdollahi-Roodsaz S., Joosten L.A.B., Roelofs M.F., Radstake T.R.D.J., Matera G., Popa C., van der Meer J.W.M., Netea M.G., van den Berg W.B. (2007). Inhibition of Toll-like Receptor 4 Breaks the Inflammatory Loop in Autoimmune Destructive Arthritis. Arthritis Rheum..

[115] Mangan M.S.J., Olhava E.J., Roush W.R., Seidel H.M., Glick G.D., Latz E. (2018). Targeting the NLRP3 Inflammasome in Inflammatory Diseases. Nat. Rev. Drug Discov..

[116] Szekanecz Z., Szamosi S., Kovács G.E., Kocsis E., Benkő S. (2019). The NLRP3 Inflammasome—Interleukin 1 Pathway as a Therapeutic Target in Gout. Arch. Biochem. Biophys..

[117] Qing Y.-F., Zhang Q.-B., Zhou J.-G. (2013). Innate Immunity Functional Gene Polymorphisms and Gout Susceptibility. Gene.

[118] Demidowich A.P., Davis A.I., Dedhia N., Yanovski J.A. (2016). Colchicine to Decrease NLRP3-Activated Inflammation and Improve Obesity-Related Metabolic Dysregulation. Med. Hypotheses.

