# Supplementary material for: β Boswellic Acid Blocks Articular Innate Immune Responses: An In Silico and In Vitro Approach to Traditional Medicine
Source: Antioxidants (Basel). 2023 Feb 3;12(2):371. doi: 10.3390/antiox12020371 (PMC9952103; doi:10.3390/antiox12020371)
Supplement: Supplementary file 1 [file antioxidants-12-00371-s001.zip › antioxidants-2084312-Supplementary Figures.pdf]

Supplementary Figures

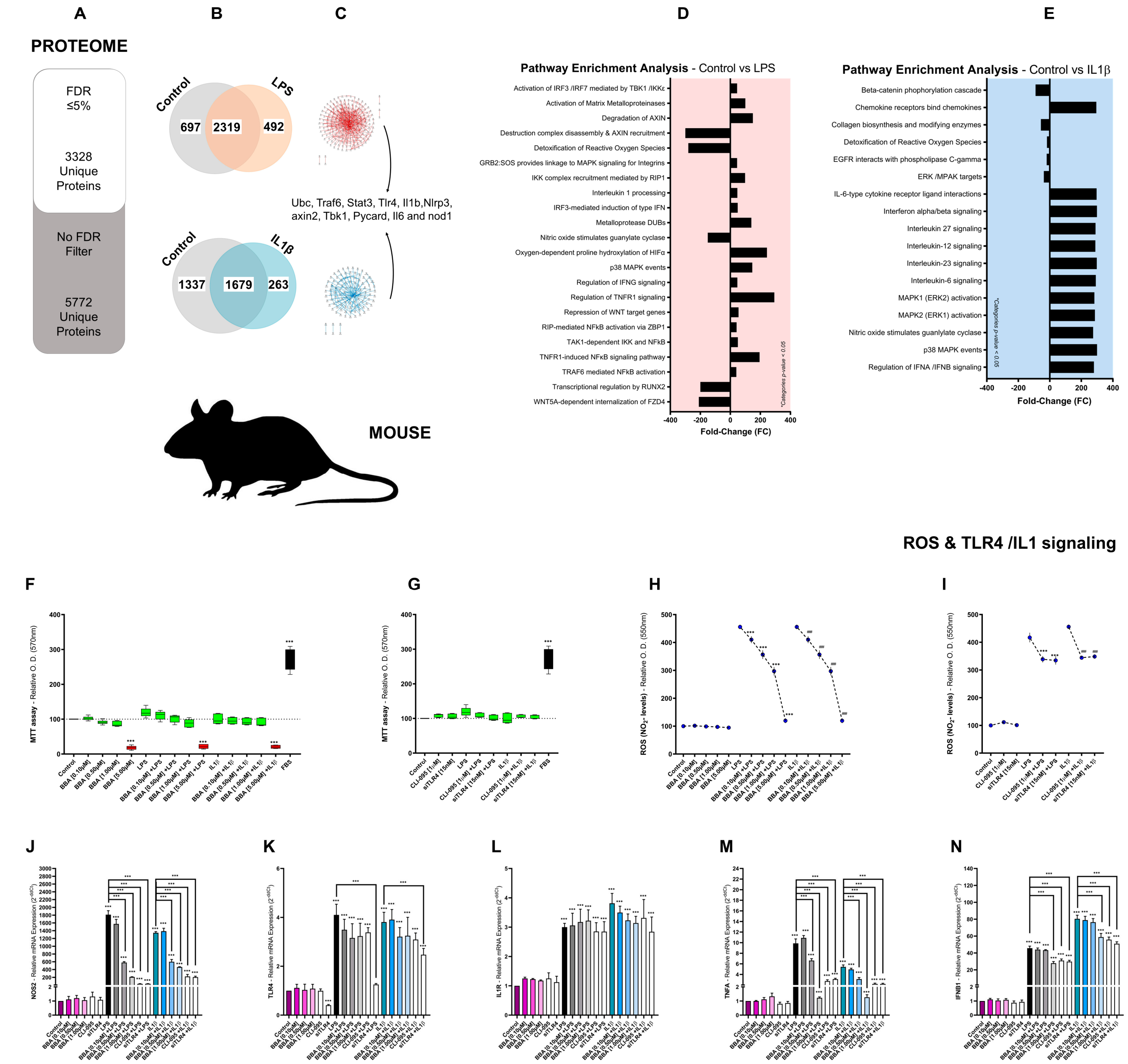

Figure S1. BBA effects on the proteome and transcriptome of TLR4 /IL1R-mediated innate immune responses in mouse ATDC5 chondrocytes: (**A-E**) Proteome profile by DDA MALDI-TOFF of mice ATDC5 chondrocytes stimulated with LPS [100ng/ml] or IL1β [0.1ng/ml] for 48h. Independent biological replicas (n=3) were normalized (control) and expressed as the mean ±SEM; (**A**) Mice proteome profile of non-filtered (FDR≤0% = 5772) and filtered (FDR≤5% = 3328) cellular proteins; (**B**) Venn diagram (FDR≤5%) of TLR4 (LPS) /IL1R (IL1β)-activated and non-activated (Control) mice ATDC5 chondrocytes proteome; (**C**) Interactome plot (FDR≤5%) of TLR4 /IL1R-activated mice ATDC5 chondrocytes proteome; (**D-E**) Pathway Enrichment Analysis (FDR≤5%; fold-change) of TLR4 /IL1R-activated mice ATDC5 chondrocytes proteome. (**F-N**) Mouse ATDC5 chondrocytes were treated with BBA [0.10-5.00μM], CLI-095 [1μM] or siTLR4 [15nM] for 24h and cotreated with LPS [100ng/ml] or IL1β [0.1ng/ml] for another 48h. Independent biological replicas (n=6) were normalized (control) and expressed as the mean ±SEM; (**F, G**) MTT cell viability assay in ATDC5 chondrocytes displaying cell inviable doses (red), cell-viable doses (green) and FBS as a positive control; (**H, I**) Griess - ROS assay

measuring NO_{2^-} levels in ATDC5 chondrocytes; (**J-N**): RT-qPCR gene expression (mRNA) of NOS2, TLR4, IL1R, TNFα and IFNB1 in ATDC5 chondrocytes.

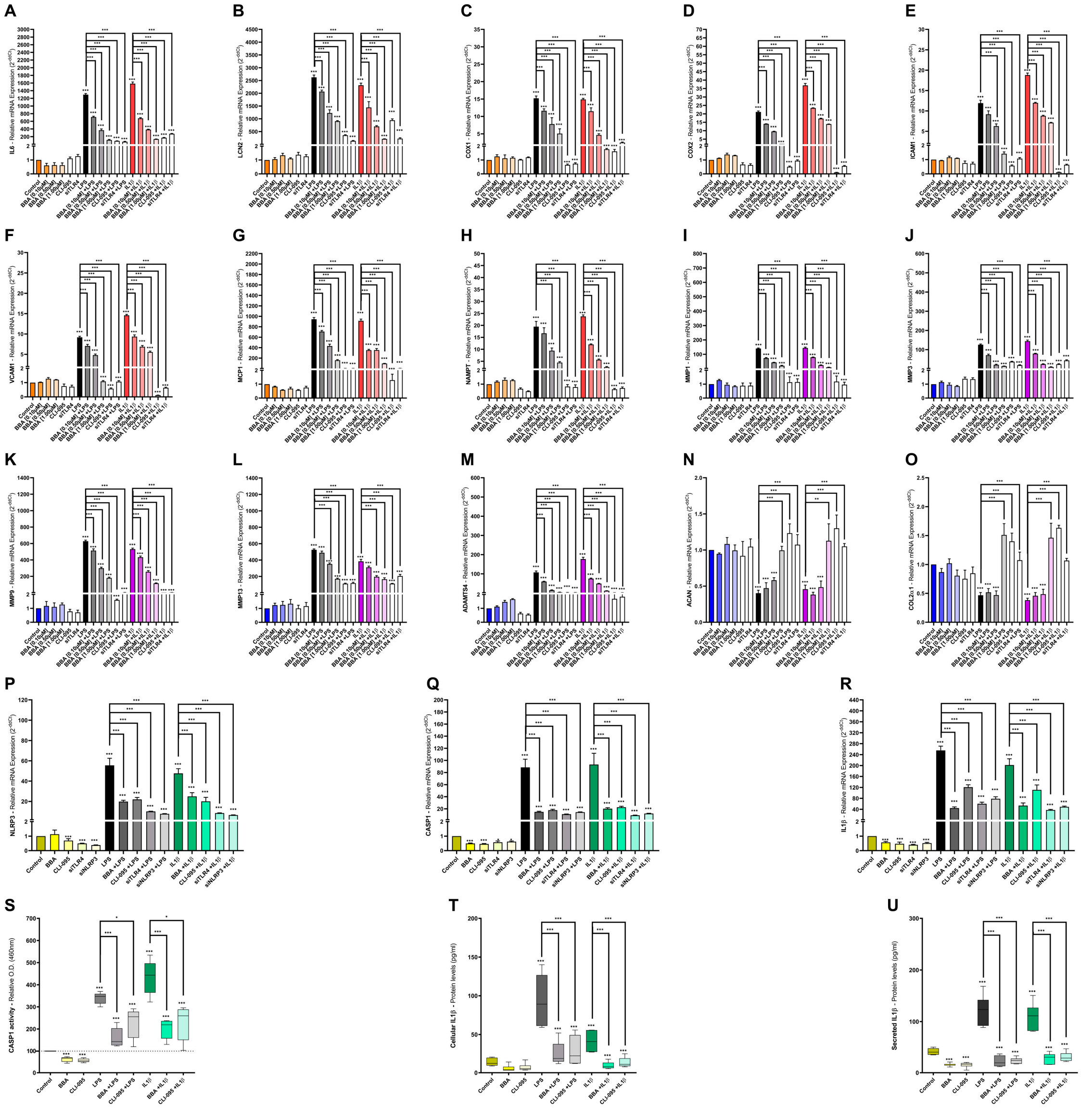

Figure S2. BBA effects on the transcriptome of TLR4 /IL1R-mediated innate immune responses in mouse ATDC5 chondrocytes: (**A-U**) Mouse ATDC5 chondrocytes were treated with BBA [0.10-5.00μM], CLI-095 [1μM] or siTLR4 [15nM] for 24h and cotreated with LPS [100ng/ml] or IL1β [0.1ng/ml] for another 48h. Independent biological replicas (n=6) were normalized (control) and expressed as the mean ±SEM. (**A-R**) RT-qPCR gene expression (mRNA) of IL6, LCN2, COX1, COX2, ICAM1, VCAM1, MCP1, NAMPT, MMP-1, -3, -9, -13, ADAMTS4, ACAN, COL2α1, NLRP3, CASP1 and IL1β in ATDC5 chondrocytes; (**S**) CASP1 activity (pNa reaction) assay in mice ATDC5 chondrocytes; (**T-U**): ELISA of cellular (**T**) and secreted (**U**) IL1β protein (pg/ml) in mice ATDC5 chondrocytes.
